# Lithium-Free Metal Fatty Acid-Based Electrolytes for Quasi-Solid-State Photosupercapacitors

**DOI:** 10.1021/acsomega.5c10959

**Published:** 2026-04-01

**Authors:** Emine Karagoz, Cemre Oztop, Deren Aydin, Cigdem Tuc Altaf, Nurdan Demirci Sankir, Mehmet Sankir

**Affiliations:** † Micro and Nanotechnology Graduate Program, 64013 TOBB University of Economics and Technology, Sogutozu Caddesi No 43 Sogutozu, 06560 Ankara, Turkey; ‡ Department of Materials Science and Nanotechnology Engineering, TOBB University of Economics and Technology, Sogutozu Caddesi No 43 Sogutozu, 06560 Ankara, Turkey; § Fotontek Optical Communication Ltd. Sti., Aso Technopark Ahi Evran Osb, Erkunt Avenue No: 3 Office No: 114, 06935 Sincan/Ankara, Turkey

## Abstract

Novel lithium-free gel metal fatty acid (M:FA, M = Co, Cu, and Fe) electrolytes were prepared for use in photosupercapacitor (PSC) applications. Cyclic voltammetry (CV) analysis revealed that the device using the Fe:FA gel electrolyte had a superior specific capacitance (*C*_p_) of 644 Fg^–1^ at 1 mVs^–1^. However, for higher scan rates, the Cu:FA gel electrolyte-based PSC performed better compared to the other M:FA. Galvanostatic charge–discharge (GCD) experiments also provided values close to the moderate scan rates of CV experiments, with *C*_p_ values obtained from GCD measurements under 20 Ag^–1^ and illumination conditions being 318, 265, and 252 Fg^–1^ for Cu, Co, and Fe:FA, respectively. Besides, to assess cyclic stability, devices were tested at a voltage of 1 V and a current density of 60 Ag^-1^ for 10,000 cycles. The *C*_p_ retention was found to be 67%, 117%, and 55% of the original capacitance for Cu, Co, and Fe:FA PSCs, respectively, with almost 100% Coulombic efficiency for all PSCs. In line with all of these results, it was concluded that Cu and Fe:FA-based PSC devices combine high performance and durability with low cost and abundance in nature as an alternative to lithium-based electrolytes and shed light on future studies.

## Graphical abstract

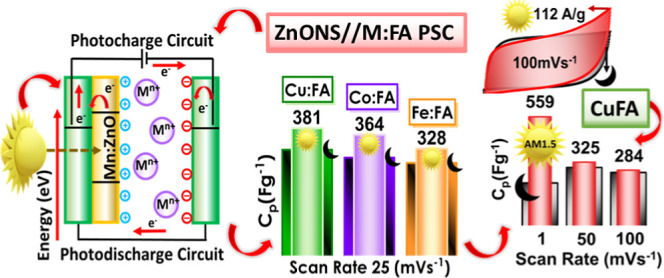

## 1 Introduction

As electrical devices have become more and more prevalent in our lives, innovative electrochemical energy conversion and storage devices have become increasingly important to meet the power needs of these devices. Electrochemical energy conversion and storage devices can be considered in a wide range of areas, from hydrogen technologies to batteries and supercapacitor devices.^1–8^ In recent years, conversion and storage devices that utilize solar energy in electrochemical systems have come to the fore.^9–14^ These devices are advantageous and cost-effective because they enable direct conversion of solar energy into electricity or other forms, thus allowing storage. These devices utilize solution-based production methods, which are also used in the production of thin-film solar cells, and thus offer the advantages of compactness, lightweight design, and low cost.^15–18^ For an electrochemical energy storage device, electrode properties are the key parameters of electrochemical performance, so researchers are dedicated to preparing highly efficient electrodes.^19^

In recent years, researchers have not only focused on the preparation of new electrode materials for energy storage applications but also conducted extensive research on the study and development of electrolytes.^20^ Electrolytes are crucial components that play a key role in determining the energy density and safety of supercapacitors, acting as the primary ionic conductor between two electrodes and facilitating the flow of the ionic current. Ion transport is crucial because it affects specific capacitance, operating voltage, and cycle stability.^20–22^ The performance of an electrolyte is determined by various factors, including ionic conductivity, operating temperature, voltage range, electrochemical window, stability, and safety. Furthermore, the high electrochemical and chemical stability of the electrolyte significantly extends the supercapacitor’s long service life.^20,21^ Traditional aqueous and organic electrolytes offer high ionic conductivity and ease of use, but they often cause problems, such as evaporation or corrosion, when used with strong acids or bases. To overcome these challenges, recent research has focused on polymer or gel electrolytes to improve mechanical performance and ionic mobility.^23^ Gel electrolytes, consisting of an electrolyte salt, a polymer matrix, and a plasticizer, have emerged as important components in solid-state supercapacitors for energy storage devices with their high conductivity, mechanical strength, thermal, and electrochemical properties.^21,22^ Commonly used polymers include polyoxyethylene, polyether ether ketone, poly­(vinyl alcohol) (PVA), polyvinyl chloride, polyacrylamide, poly­(acrylic acid), poly­(ethylene oxide), polyvinylidene fluoride, potassium polyacrylate, and polyacrylamide.^20,22,24^ PVA is an ideal material due to its simple preparation process, high chemical stability, mechanical strength, excellent compatibility, and ability to form transparent films. Furthermore, its affordability, safety, nontoxicity, high temperature and pressure resistance, and high hydrophilicity make it widely used in energy storage devices.^20,21,25,26^

Fatty acid salts have not generally been used directly as electrodes in supercapacitors and battery applications, but their use as versatile precursors for metal oxides or carbon-based electrode materials has been reported previously.^27–29^ Recently, da Silva et al. reported the eutectic mixtures of sodium salt and fatty acids as electrolytes for supercapacitors.^30^ In another study, Arjunkumar et al. produced supercapacitors using pure green leaves of *Anisomeles malabarica* using the filamentation method (flame + deposition) and castor oil containing high amounts of ricinoleic (90%), 4% linoleic, 3% oleic, 1% stearic, and <1% linolenic fatty acids having 18 monounsaturated carbon chains. They found the specific capacitance (*C*_p_) value as 280.8 mFcm^–2^ in the supercapacitor using KOH/PVA as the electrolyte.^31^ De et al. synthesized stable monodisperse silver nanoparticles using an extract from the *Myristica fragrans*­(nutmeg) plant containing fatty acids. The electrochemical potential of silver nanoparticles was measured using cyclic voltammetry to evaluate their supercapacitor applications, and the *C*_p_ of the device was 21 Fg^-1^.^32^

Although there are works on integration of the fatty acid to the electrode materials in supercapacitors, there is no previous study reporting direct usage of M:FA-based electrolytes for a solar rechargeable energy storage device. In this work, we used metal fatty acid salts (M:FA, M = Co, Cu, and Fe) and PVA as the gel electrolyte for a photosupercapacitor. To ensure high performance, we used Mn-doped ZnO (Mn:ZnONS) as a photoactive electrode. As a result, we created a new-generation electrochemical energy storage device that can be charged directly by sunlight and does not contain lithium. The performance of naturally abundant metal fatty acid-based electrodes, such as copper and iron, will be a significant step forward for new devices in this field.

## 2 Experimental Section

### 2.1 Materials

Zinc acetate dihydrate (Zn­(CH_3_COO)_2_·2H_2_O, Sigma-Aldrich, 99.5% purity), urea crystal ((NH_2_)_2_CO, Isolab-ASC ≥99%), manganese­(II) sulfate monohydrate (MnSO_4_.H_2_O, Sigma-Aldrich ReagentPlus ≥99%), poly­(vinyl alcohol) (PVA, Sigma-Aldrich, 87–90%), acetic acid (Sigma-Aldrich, 99.8–100.5%), fluorine-doped tin oxide (FTO, Rs < 10 Ω, 2.5 cm × 7.5 cm × 0.22 cm), and fatty acid metal copper (Cu), cobalt (Co), and iron (Fe) were used.

### 2.2 Preparation of the Photoelectrode

A 10% manganese-doped and 3D zinc oxide nanosheet (Mn:ZnONS) was synthesized by the chemical bath deposition (CBD) method. 45 mM zinc acetate dihydrate, 1 M urea, 5 mM manganese­(II) sulfate monohydrate, and 200 mL of distilled water were mixed until homogeneous. Acetic acid was added, until the pH value of the solution became 4.8. The solution taken into a borosilicate bottle was heat-treated at 80 °C for 3 h. The resulting powder was filtered and dried at 50 °C for 2 days. Mn:ZnONS powder was sonicated (5h-53 Hz) with isopropyl alcohol and butanol and then dropped onto the FTO substrate (0.9 × 1.25 cm^2^). The thin film was calcined at 400 °C for 1 h.

### 2.3 Preparation of the Electrolyte

A semisolid PVA-metal-doped fatty acid (PVA-M:FA) gel electrolyte was used as the electrolyte. 0.5 g of metal (Cu, Co, and Fe) doped fatty acid, 1 g of PVA, and 10 mL of pure water were boiled in an 85 °C water bath for 4 h by stirring. Three electrolytes were prepared separately as Cu:FA, Co:FA, and Fe:FA.

### 2.4 Preparation of the Photosupercapacitor Device

A single electrode made of Mn:ZnONS was prepared on a FTO substrate. The FTO substrates were cut to dimensions of 1.86 × 1.25 cm^2^ and covered with nonmarking tape, leaving a 0.6 × 0.4 cm^2^ area exposed. The Mn:ZnONS electrode was dropped into this area at a concentration of 0.3 mg ± 0.1. To reduce the margin of error in mass measurement, weighings were taken 4–5 times before and after the dropping. The frame was made of stainless steel and carbon paper with the electrode area removed from the center. PSCs were closed with M:FA drops between each one and are secured with screws in the following order: ABS, FTO//Mn:ZnONS, frame stainless steel, frame carbon paper, filter paper, carbon paper, stainless steel, FTO, and ABS. Three different photosupercapacitor devices were produced with manganese-doped zinc oxide and different metal fatty acids. Photosupercapacitors fabricated using a Mn:ZnONS thin film and fatty acid are coded as Cu:FA PSC, Co:FA PSC, and Fe:FA PSC. Figure 1 summarizes the device production steps schematically.

**Figure 1:**
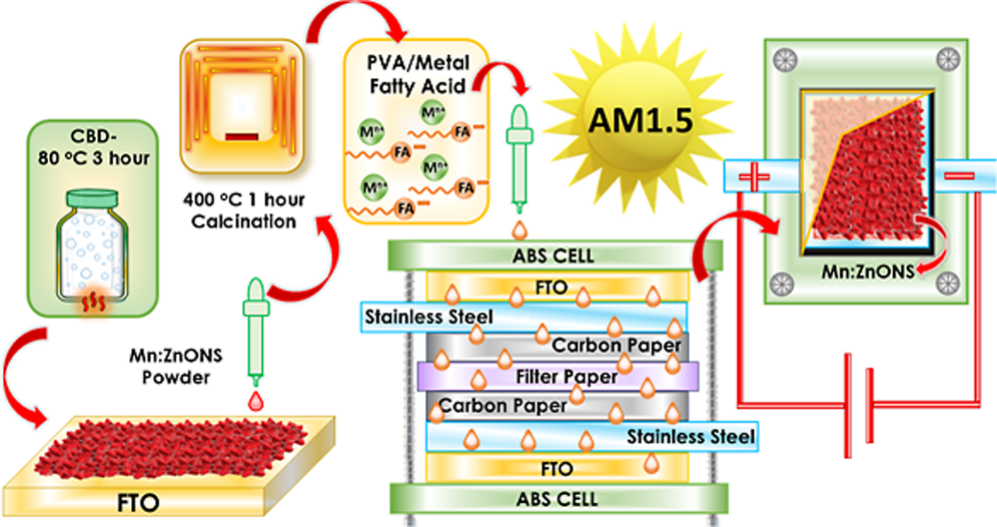
Mn:ZnONS-M/FA PSC production process and representative representation.

### 2.5 Characterization

Scanning electron microscopy (SEM), UV light source­(FemtoTera), energy-dispersive X-ray (EDS), ultraviolet–visible spectroscopy (UV–vis, PerkinElmer-650S), X-ray diffraction (XRD/Cu–K α radiation (λ = 1.54 Å), a simulated sunlight light source (AM 1.5, 100 mW/cm^2^//Lot Oriel-150 W xenon lamp), Brunauer–Emmett–Teller (BET-NOVA 1000e-Quantachrome), cyclic voltammetry (CV) and galvanostatic charge–discharge (GCD, Gamry Interface 5000), and PAR (Princeton Applied Research/PMC-1000), Ametek, were used for characterization.

### 2.6 Electrochemical Performance

For the evaluation of specific energy, specific capacitance, and specific power, galvanostatic charge–discharge (GCD) curves were recorded at various current densities at 6, 8, and 10 Ag^-1^. All measurements were made at room temperature under bright and AM1.5 conditions. The specific capacitance (*C*_p_) was calculated by CV polarization curves by employing the following equation:^33–36^

1 $$C_{p}=\frac{It_{d}}{m\Delta V}\left(F/g\right)$$

2 $$C_{p}=\frac{\int IdV}{\nu \times m\times t_{d}}\left(F/g\right)$$

here, “*I* (A)” is the discharge current, “*t*_d_ (s)” is the discharge time, “*m* (kg)” is the mass of the active electrode material, “Δ*V* (V)” is the change in voltage, and “υ (m/s)” is the scan rate of the CV. Energy density “*E* (Wh/kg)” and specific power “*P*_d_ (W/kg)” of the supercapacitor device are represented by the following equation:^33,34^

3 $$E=\frac{C_{p}\Delta V^{2}}{2\times 3.6}\left(Wh/kg\right)$$

4 $$P=\frac{E}{t_{d}}\times 3600\left(W/kg\right)$$

The Coulombic efficiency (CE) of the supercapacitor is calculated by

5 $$CE\;\left(\%\right)=\frac{\Delta t_{d}}{\Delta t_{c}}\times 100$$

Here, “Δ*t*_c_ (s)” is the charge time and “Δ*t*_d_ (s)” is the discharge time.

## 3 Results and Discussion

### 3.1 Materials Characterizations

The morphology of M:FA powders was characterized via SEM analysis (Figure 2). The Cu:FA powders had needlelike structures having tens of micrometers of length (Figure 2A). On the other hand, Co:FA powders had microplates (Figure 2B), and Fe:FA had a denser morphology compared to the Cu and Co:FA powders (Figure 2C). The SEM images and EDS analysis results of various magnifications have been given in the Supporting Information (Figures S1–S3). EDS analysis revealed that 10, 6, and 14 atomic % Cu, Co, and Fe were detected in M:FA powders, respectively.

**Figure 2:**
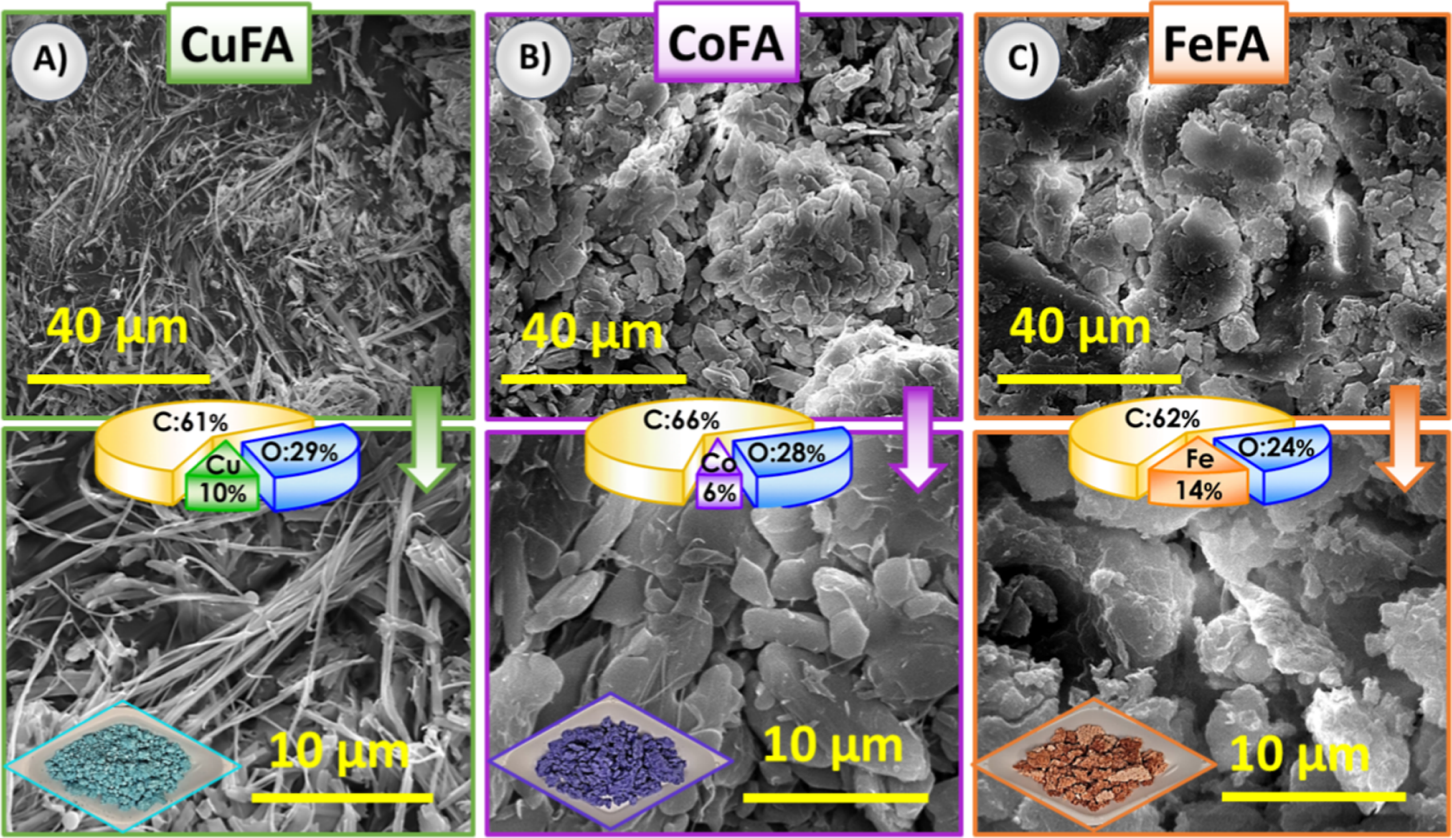
(A) SEM images and EDS of Cu:FA at different magnifications (inset: photograph of Cu:FA powder), (B) SEM images and EDS of Co:FA at different magnifications (inset: photograph of Co:FA powder), and (C) SEM images and EDS of Fe:FA at different magnifications (inset: photograph of Fe:FA powder).

The surface area of the synthesized sample was determined using the Brunauer–Emmett–Teller (BET) method via nitrogen absorption–desorption. The powder and sonicated powder samples were loaded into the adsorption cell and activated under high vacuum and a temperature of 150 °C. Ultrahigh purity nitrogen gas (N_2_: 99.998%) was used while taking the adsorption measurements. Figure 3A shows the type IV adsorption isotherm at high relative pressures. It occurs in mesopores, desorption occurs with increasing pressure, and hysteresis formation is observed.^37–39^ The surface area of the Mn:ZnONS material with high crystallinity was found to be 62 m^2^ g^–1^ before sonication and 63 m^2^ g^–1^ after sonication (Figure S4). This high surface area obtained in the electrode caused a more capacitive effect. The pore size of the synthesized sample is shown in Figure 3B. An obvious 20 nm peak is seen in the size distribution curve of the material, consisting predominantly of mesopores and fewer macropores. UV–vis absorption spectra were measured to investigate the optical properties of the Mn:ZnONS thin film. Figure 3C shows the optical absorption spectra of the Mn-doped ZnONS sample measured at room temperature in the wavelength range from 300 to 800 nm. The estimated band gap of Mn:ZnONS at 375 nm wavelength is 3.31 eV. This value is the same as the band gap of bulk ZnO (3.31 eV) in the visible range and acts as an effective photoactive material.^40^ X-ray diffraction (XRD) measurements were performed at room temperature in the range 10–80° with a step of 0.013° (Figure 3D). The peaks at 31.39°, 34.40°, and 36.44° shown in the XRD spectra correspond to the orientations of wurtzite ZnO (100), (002), and (101), respectively (PDF-01-079-0205). The peaks at 13.26°, 33.16°, and 69.12° correspond to the orientations of cubic Mn_2_O_3_ (110) and (222), respectively (PDF-01-076-0150). With the doping of Mn into the ZnO crystal, a sharp diffraction plane (222) is formed next to the (002) plane. The crystal size was calculated from the Scherrer equation.^41^

6 $$D=\frac{k\lambda }{\beta Cos\theta }$$

**Figure 3:**
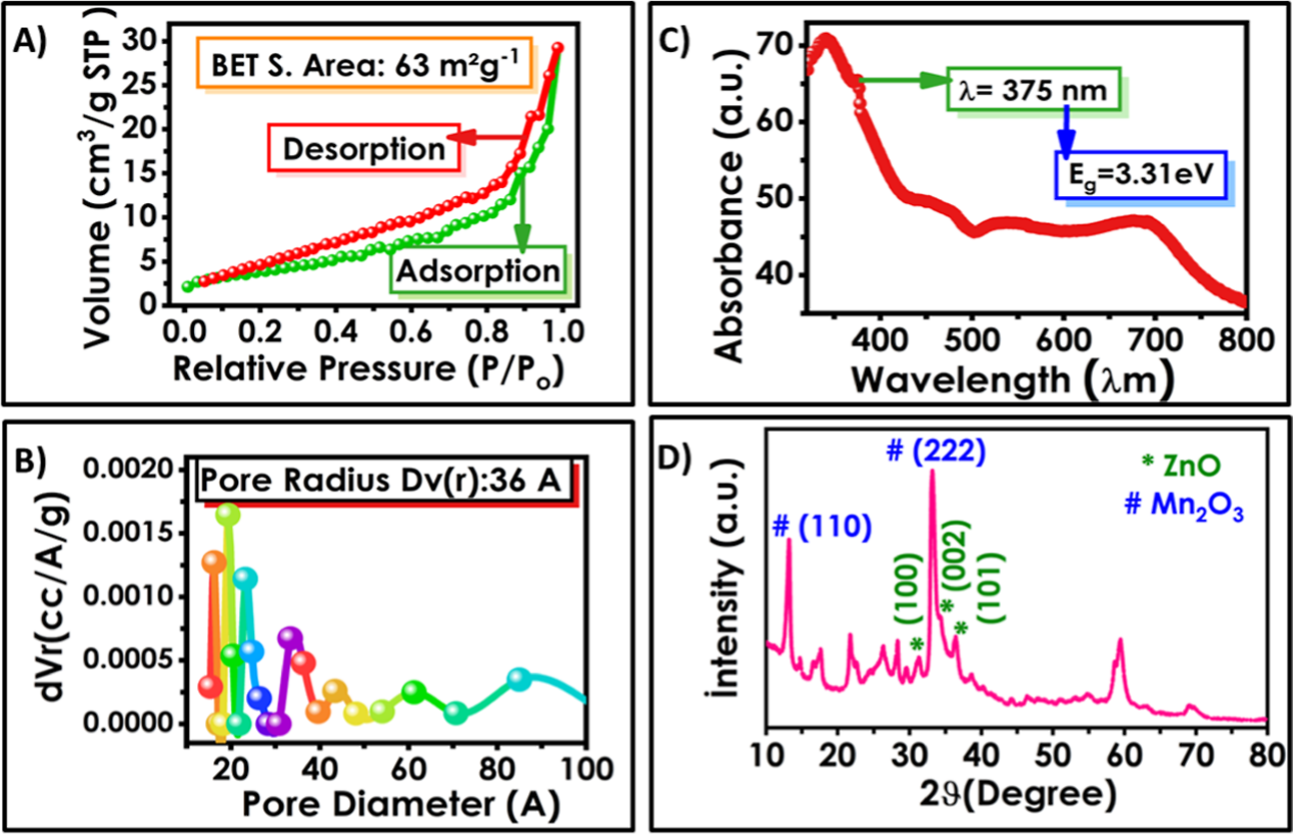
(A,B) BET surface area nitrogen adsorption–desorption isotherms, (C) optical absorbance spectra, and (D) X-ray Diffraction (XRD) of Mn:ZnONS.

Here, *k* is the shape factor, λ is the wavelength of the incident X-ray, β is the full width at half-maximum (fwhm) of the XRD peak in radians, which was calculated as 0.699°, and 2θ was calculated as 33.16°. According to eq 6, the calculated crystallite size of the Mn-doped ZnO is 12 nm, which is in agreement with the literature.^42,43^

SEM analyses of Mn:ZnONS were performed at different magnifications before and after sonication, which is used to prepare the ink to make the photoactive electrode (Figure 4). The high surface area is also supported by SEM images. From the SEM images, it can be clearly seen that the ZnO nanosheets formed microballs with a nanostructure. The dense nanosheets resulted in a large surface area. SEM images of Mn:ZnONS powder and thin film electrodes with various magnifications and energy dispersive X-ray spectroscopy (EDS) analysis have been given in Figures S5 and S6. EDS results and mapping analysis of the Mn/ZnO thin film are presented in Figures 4C and 4F, respectively. It has been observed that the presence of Mn, Zn, and O elements and their atomic percentages are estimated as 1%, 37%, and 62%, respectively. Mapping results clearly show that Mn, Zn, and O elements are uniformly distributed throughout the thin film.

**Figure 4:**
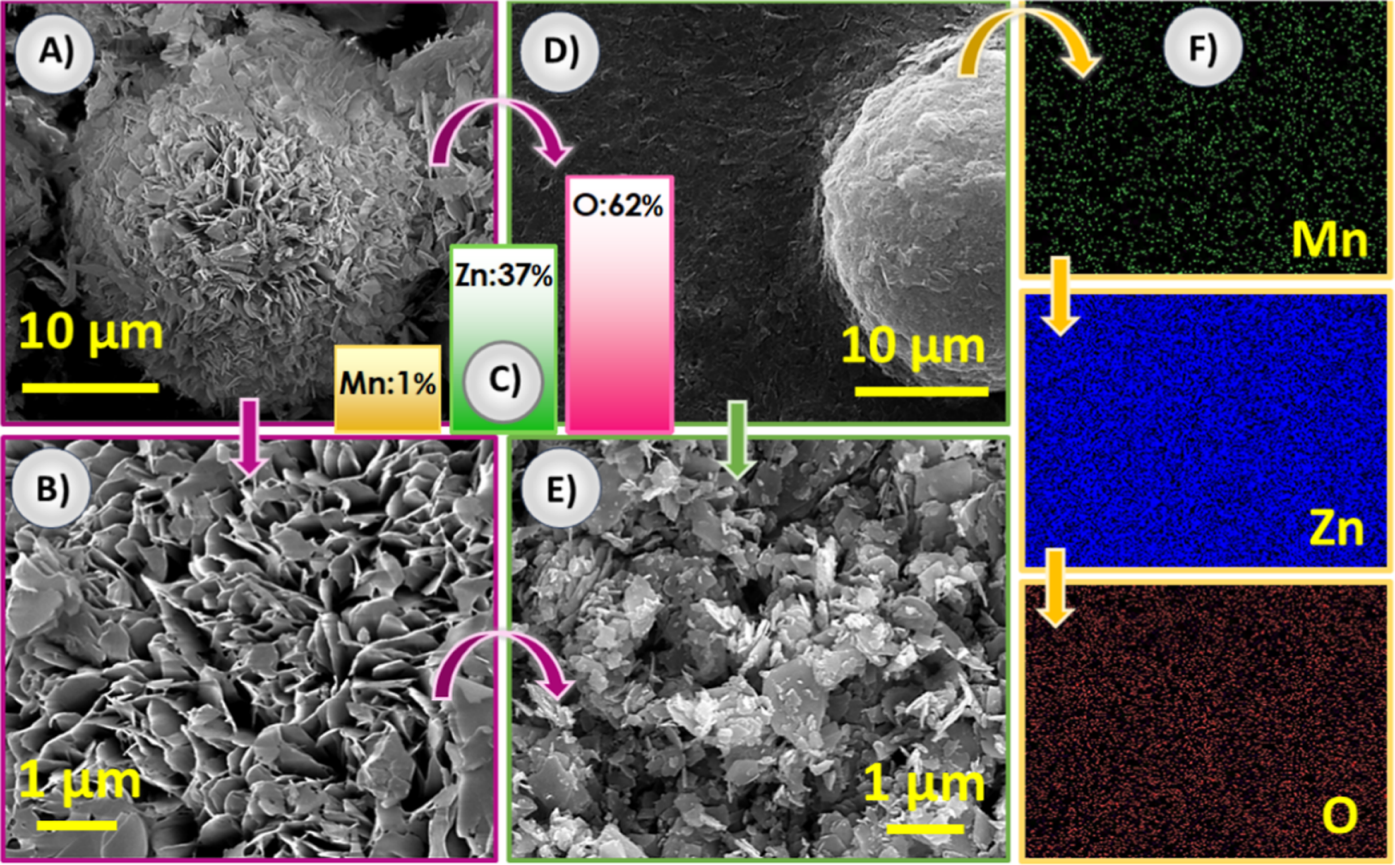
(A,B) SEM analysis at 7,000 and 40,000 magnifications (before sonication), (C) EDS (after sonication), (D,E) SEM images at 7,000 and 40,000 magnifications (after sonication), and (F) elemental mapping images (before sonication) of Mn:ZnONS powder.

### 3.2 Electrochemical Measurements

Doping transition metal ZnO with Mn improves its electrical and optical properties and offers significant potential for tunable photoelectronic applications.^44,45^ Mn-doped ZnO can easily accommodate Mn^2+^ (0.74 Å) and Zn^2+^ (0.67 Å) in the ZnO lattice structure due to the small difference in ionic radii and offers an advantage because of its half-filled 3d orbitals.^45,46^ Mn doping of ZnO crystals creates redox centers that engage in electron transfer reactions and facilitates faradaic reactions on or near the surface of the electrode material. Furthermore, Mn doping increases electrical conductivity by creating oxygen vacancies within the ZnO structure and provides additional active sites for redox interactions with electrolyte ions.^47^ In our previous study, manganese doping of ZnONR was performed, its detailed characterization was carried out, and photodetector tests were conducted. It was clearly demonstrated that manganese doping significantly improved the photodetector performance. Therefore, in this study, ZnONS with a high surface area were doped with manganese as a photoactive material. Cyclic voltammetry (CV) has been used to analyze PSC devices in a potential range of 0 to 1.1 V at various scan rates (Figures 5A,B and S7–S10). All samples were analyzed in the dark (Figure 5A) and under AM1.5 illumination (Figure 5B). It has been observed that the current density and, therefore, the specific capacitance (*C*_p_) were enhanced with light. This enhancement was 17%, 20%, and 13% for Cu, Co, and Fe:FA-based PSC devices, respectively. The highest *C*_p_ of 381 Fg^-^^1^has been calculated for the Cu:FA-containing electrolyte at 25 mVs^–1^ under illumination (Figure 5C).

**Figure 5:**
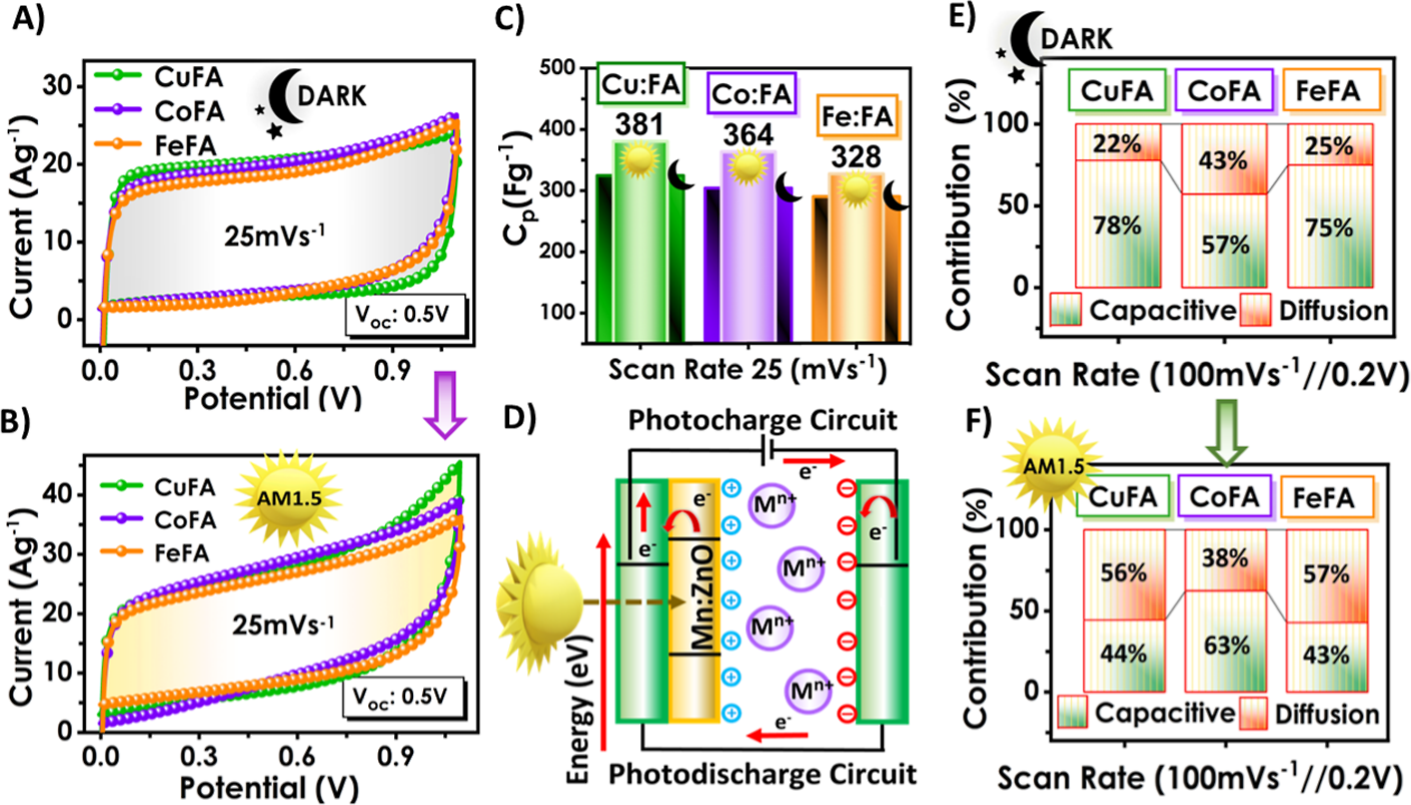
(A,B) CV graph at 25 mVs^–1^ scanning rate and in the dark and AM1.5 light, (C) *C*_p_ graph at 25 mVs^–1^ scanning rate and in the dark and AM1.5 light, (D) schematic representation of the working principle of the photosupercapacitor, and (E,F) capacitive and diffusive contributions at different scan rates under dark and AM1.5 light conditions of M/FA-based PSCs.

At low scan rates, the ions in the electrolyte can reach both the outer and inner surfaces of the electrode and participate in Faradaic reactions. However, at higher scan rates, the energy storage process is limited to the outer surface, which reduces the specific capacity. Therefore, increasing the scan rate can reduce the specific capacity of the electrode.^48^ The dark CV curves for M/FA-based PSC devices are almost rectangular, indicating an electric double-layer capacitance (EDLC) charge storage process. Under AM1.5 light, the redox peaks in the CV curve show that the interaction with metal ions in the M:FA/PVA electrolyte occurring in the PSC and the pseudocapacitance resulting from the insertion/extraction charge storage process are due to photoinduced charge carriers. These carriers increase charge dissociation, reduce charge transfer resistance, facilitate charge transport, and reduce interface resistance.^49^ During photocharging, photogenerated electrons produced by sunlight striking the photoactive Mn:ZnONS electrode flow from the conductive electrode to the counter electrode, causing this electrode to become negatively charged. Thus, charge accumulation begins in the device (Figure 5D).

Dunn’s analysis was performed to understand where the capacitive effect occurs due to EDLC or diffusion mechanisms. Dunn’s equation relates the total current at a given potential to the scan rate.

7 $$i\left(V\right)=k_{1}\nu +k_{2}\nu^{1/2}$$

Here, *k*_1_(*v*) represents the capacitive-controlled current and is obtained from the slope of the graph. *k*_2_(*v* ^1/2^) represents the diffusion-controlled current and is found from the *y*-intercept of the graph.^50,51^

8 $$i\left(V\right)/\nu^{1/2}=k_{1}\nu^{1/2}+k_{2}$$

Calculations were performed for M:FA PSCs at different scan rates (1–100 mVs^–1^) and voltages (0.2 V–1 V). The capacitive and diffusion contributions of M:FA PSCs at a 100 mVs^–1^ scan rate and 0.2 V voltage in dark and illumination conditions are presented in Figure 5E,F. As a general trend, for dark measurements, the capacitive contribution to the *C*_p_ was superior to the diffusive part. Under illumination, except Co:FA, other PSC devices showed an increase in diffusive contribution, which could be due to the internal electric field occurring with photogenerated charges and activating additional redox sites. On the other hand, for the Co:FA, the increase in capacitive contribution could be due to the surface potential shift with photogenerated charges, which may enhance both electric double-layer formation and surface pseudocapacitance. Figures S11–S13 show the contribution ratios in dark and illumination conditions for various scan rates. At low scan rates, diffusion control is observed, while at high scan rates, capacitive control is observed. In the dark, capacitance values increased from 26% to 78% for Cu:FA, from 12% to 57% for Co:FA, and from 23% to 75% for Fe:FA with an increasing scan rate.

The decrease in diffusion current with increasing scan speed indicates that M:FA electrolyte ions have limited time to diffuse to the ZnO photocapacitive electrodes.^50^ In the illuminance, with increasing scan speed, the capacitive effect increased from 29% to 44% for Cu:FA, from 54% to 63% for Co:FA, and from 7% to 43% for Fe:FA. The *b* value is used to investigate the mechanism behind the device’s charge storage capacity. For an ideal supercapacitor, *b* should be equal to 1, while for pure battery-grade materials, it should be equal to 0.5. The *b* values were obtained by linearly fitting the CV curve of the device shown in Figures S11–S13. The *b* value, which lies between 0.5 and 1, which is within the range of batteries and supercapacitors, indicates the presence of both diffusional and capacitive charge storage mechanisms in the supercapacitor device.^52^

To investigate the effect of different metal fatty acid electrolytes, CV graphs measured at different scan rates under AM1.5 illumination conditions were examined (Figure 6). Among the three M:FA electrolytes, the Cu:FA PSC exhibited the highest current density, demonstrating superior capacitive behavior and redox activity. All M:FA PSCs showed good rate performance, maintaining the current response shape of the CV curve in both dark and light conditions. With illumination, all electrodes, especially Cu:FA PSC, exhibited photosensitive properties by significantly increasing the current density and area under the curve. This increase under illumination is related to the increase in photogenerated charge carrier activity, which enhances redox processes and overall energy storage performance.^53^ Furthermore, to better elucidate the role of metal fatty acid electrolytes, CV measurements of the Cu:FA PSC without electrodes were taken and are presented in Figure S7B. In the absence of the Mn:ZnO electrode, it still exhibited capacitive behavior and redox activity but showed superior performance in the presence of the electrode. The compatibility of the Mn:ZnO electrode and M:FA electrolyte provided the maximum active surface area for electrochemical reactions and exhibited electrical double-layer capacitor (EDLC) behavior. As a result, charge was stored at the electrode/electrolyte interface through the physical adsorption of ions, which caused the resulting current–voltage graphs to appear as nearly rectangular shapes.^47,54^ Moreover, the shape of the curves was almost rectangular at all scanning rates, indicating low resistance and good reversibility. At a low scanning rate (1 mVs^–1^), the highest *C*_p_ value (644 Fg^-^^1^) was observed in the device using the Fe:FA gel electrolyte, but when all scanning rates were evaluated, the best performance was obtained with the Cu:FA PSC. It reached 559 Fg^-^^1^ at a low scanning rate and 284 Fg^-^^1^ at a high scanning rate. This result indicated that low-cost and Earth-abundant copper-based fatty acids can be integrated into PSC devices successfully.

**Figure 6:**
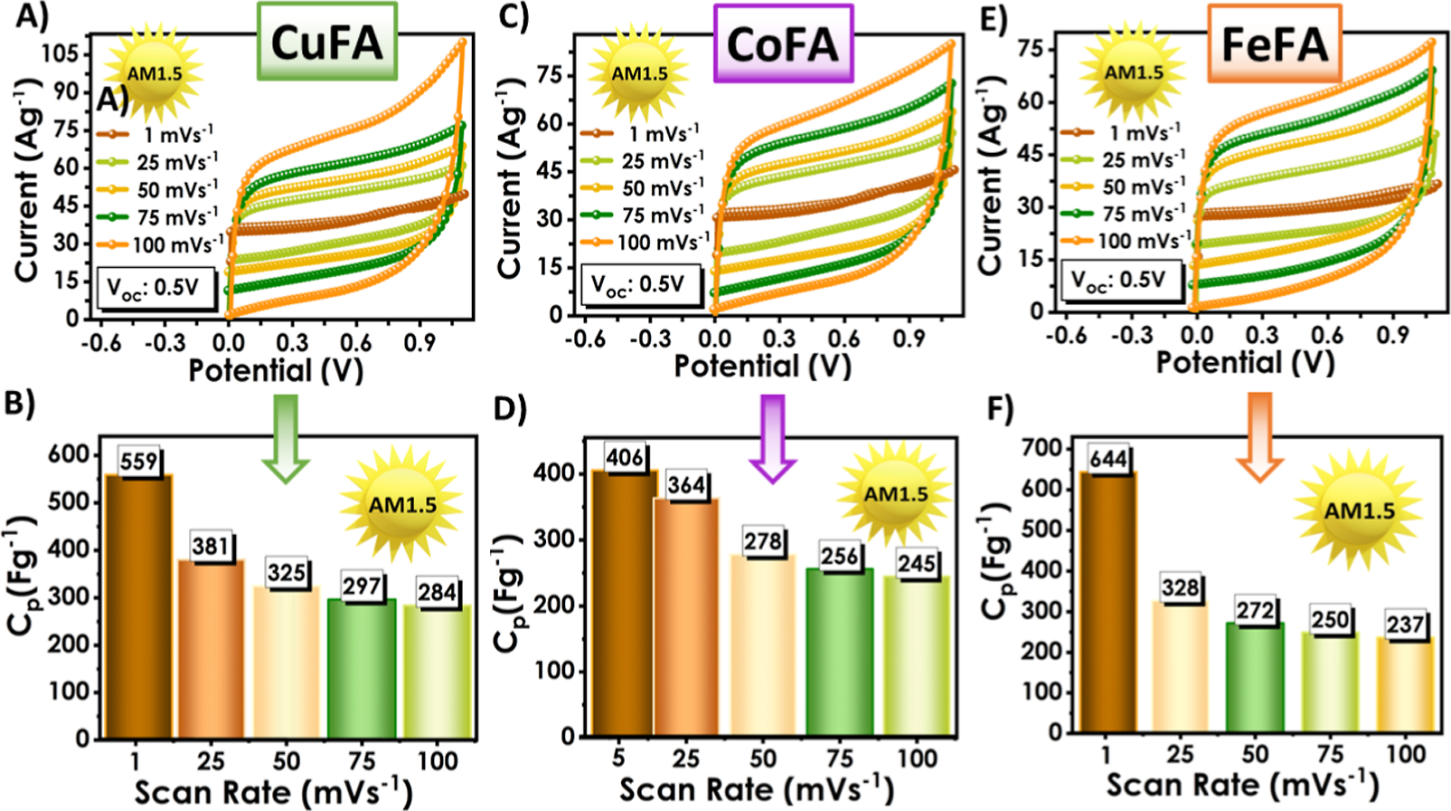
(A–C) CV analysis and (D–F) *C*_p_ analysis of M:FA-based PSC devices under various scanning rates and AM1.5 light.

Further electrochemical investigation was performed via galvanostatic charge–discharge (GCD) analysis in the dark and under illumination (Figure 7). At 20 Ag^-^^1^ current density, both charge and discharge times were increased by illumination (Figure 7A,B). A triangular shape (isosceles triangle) for charge/discharge graphs indicates an ideal capacitor.^55^ Furthermore, the almost complete absence of an IR drop indicates good contact between the materials and current collectors, efficient energy dissipation, and low internal resistance.^56^ The *C*_p_ calculated from the GCD plot at 20 Ag^-^^1^ under illumination was 318, 265, and 252 Fg^-^^1^ for Cu, Co, and Fe:FA electrolyte-based PSCs, respectively (Tables S1–S3). Besides, the highest *C*_p_ of 336 Fg^-^^1^ has been calculated for the Cu:FA at 10 Ag^-^^1^ current density and under illumination.

**Figure 7:**
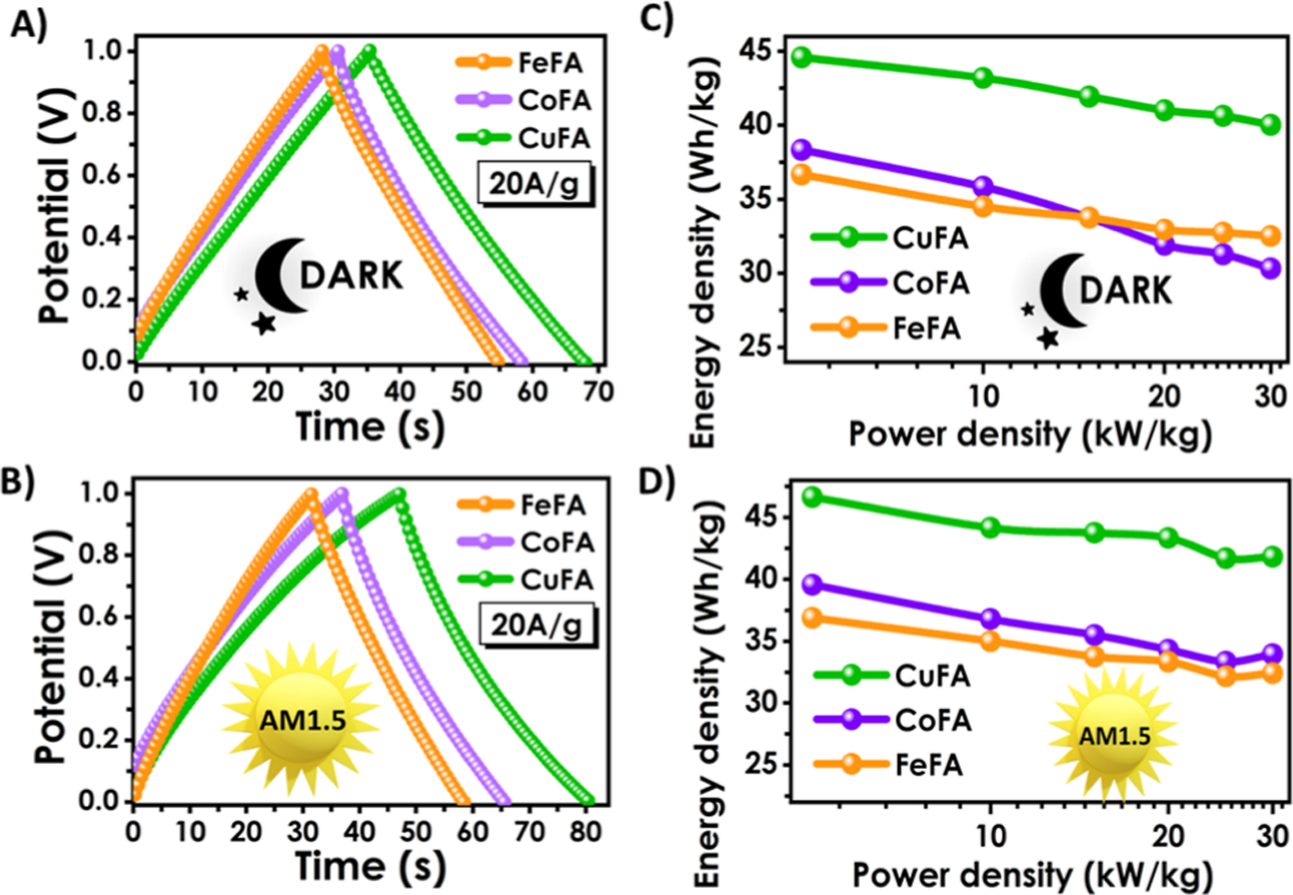
(A,B) GCD results and (C,D) Ragone plot graphs of M:FA-based PSC devices under various scanning rates and AM1.5 light.

GCD results show that the Cu:FA PSC has the best match between the electrode and electrolyte, and Cu:FA exhibits the best ionic conductivity.

Ragone plots indicated that the Cu:FA-based electrolyte resulted in superior energy and power densities compared to both Co and Fe:FA (Figure 7C,D). In addition, the energy density values of Co and Fe fatty acid-based electrolytes, especially at high power densities, were in favor of devices using Fe-based electrolytes in the dark, while the performance of devices containing Co:FA electrolytes was better under AM1.5 illumination conditions. According to studies in the literature (Table S5), a high energy density has been obtained. This is because the resistance of the supercapacitor is minimized, while the current collection is maximized.

Linear sweep voltammetry (LSV) measurements of M:FA PSCs were taken to evaluate the current density and photoresponse (Figure S14). LSV measurements were performed in the 0–1 V range at a rate of 50 mVs^–1^ in both dark and light conditions. The highest current value was obtained in the Cu:FA PSC, followed by Fe:FA and Co:FA PSCs. The high current value in the Cu:FA PSC indicates that copper ions in the electrolyte exhibit better ionic conductivity compared to iron and cobalt ions. An increase in current values was observed in the M:FA PSC when excited by light. This is due to photogenerated charge carrier activity.

Electrochemical impedance spectroscopy (EIS) measurements were taken to better understand the electrochemical effects of the metal salt. EIS is an effective characterization method for monitoring the electrochemical behavior and changes occurring at the electrode/electrolyte interface in various electrical storage and conversion devices.^57^ Furthermore, this method is important for interpreting electrical properties from the impedance change when light is incident on a photoactive material. When light is incident on a semiconductor material, its electrochemical properties change significantly compared to measurements taken in the dark. When a semiconductor material is excited by a photon, photoexcited carriers are generated. These charge carriers affect electrical conductivity, charge transfer resistance, and capacitance, causing changes in the electrochemical impedance spectra.^58,59^

EIS measurements of Cu:FA, Co:FA, and Fe:FA PSCs were taken under darkness and AM1.5 illumination, applying an AC voltage of 5 mV amplitude in the frequency range of 100 kHz to 0.1 Hz. Nyquist plots, the primary means of visualizing electrochemical impedance spectroscopy (EIS), were plotted for each sample (Figure 8). The electrical equivalent circuit (EEC) model was used to calculate impedance data from the Nyquist plots. Analyzing the equivalent circuit model revealed the presence of two regions in the Nyquist plots. The first region is the high-frequency region consisting of a semicircle. This region represents the charge transfer resistance of the electrode due to ionic and electronic impedance resulting from charge diffusion at the electrode/electrolyte interface. The second region is the low-frequency region consisting of a curve. This region represents the diffusion resistance of ions between the electrolyte ions and the electrode pores, a consequence of the Warburg impedance (W).^60,61^

**Figure 8:**
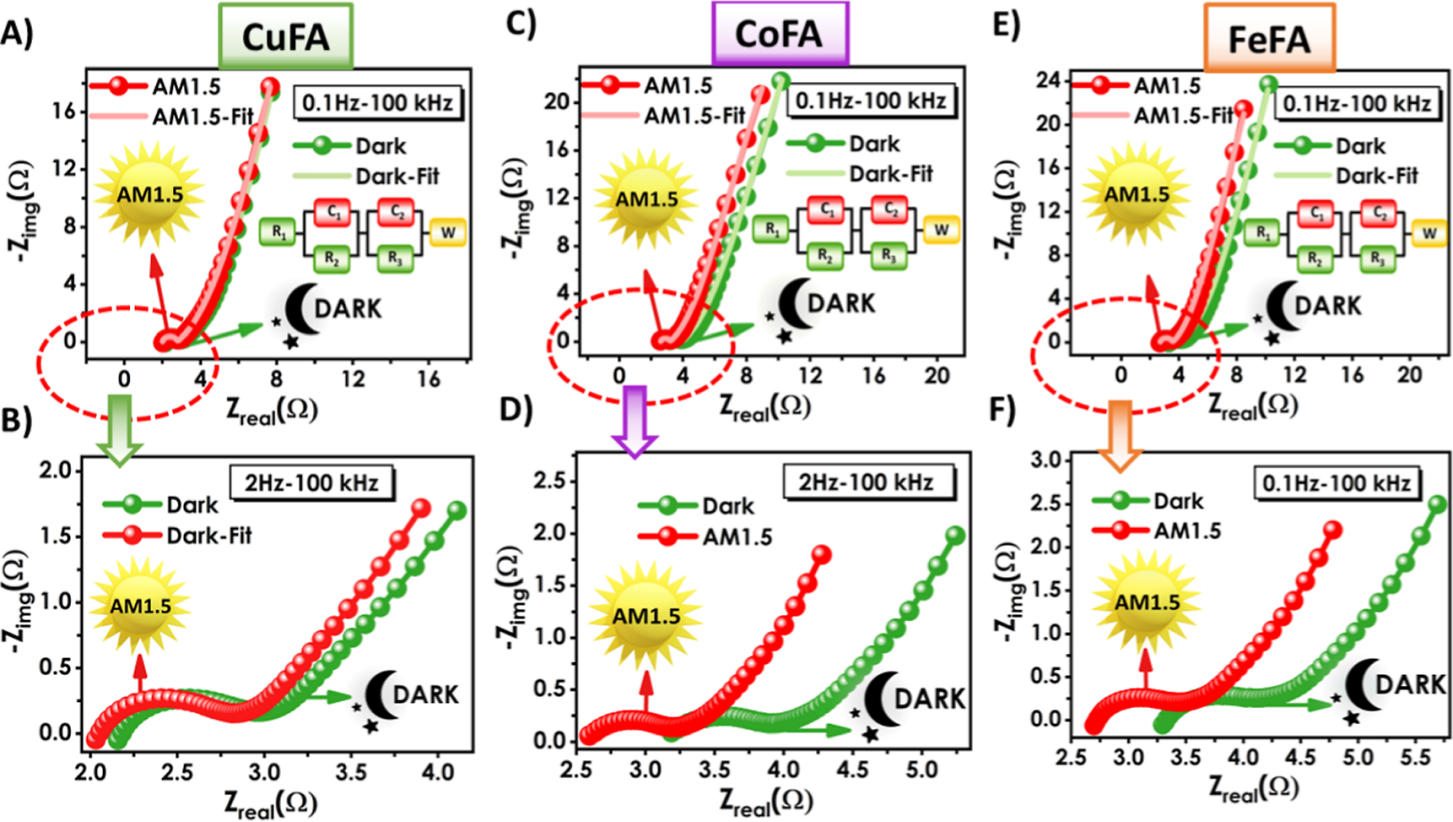
(A–F) M/FA-based PSC devices; EIS characteristics (Nyquist plots) with the fitted circuit diagram.

The equivalent circuit model solution included circuit elements *R*_1_, *R*_2_, and *C*_1_ for the capacitive region corresponding to the semicircle and *R*_3_, *C*_2_, and W for the diffusion region corresponding to the curve. Circuit data are presented in Table S4. The *x*-axis intercept corresponds to the ultralow electrolyte resistance (*R*_1_) at room temperature, indicating superior ion diffusion coefficients and high conductivity. Here, the metals (Cu, Co, and Fe) in the electrolyte create highly conductive paths within the device, effectively increasing electron mobility and reducing the overall charge transfer resistance.^62^ Under light conditions, the electrochemical behavior of the samples improved significantly compared to that observed in the dark. In the dark, the values for Cu:Fa, Co:Fa, and Fe:FA PSCs were 2.18 Ω, 3.2 Ω, and 3.3 Ω, respectively, while they decreased to 2 Ω, 2.6 Ω, and 2.7 Ω upon illumination, increasing current. Additionally, illumination with light caused a decrease in the *R*_3_ and W values in the diffusion region. Furthermore, EIS measurements were taken of M:FA PSCs without an electrode, and they were shown to exhibit good conductivity (Figure S15). The *R*_1_ values of Cu:FA, Co:FA, and Fe:FA PSCs were 3.38 Ω, 5.38 Ω, and 4.8 Ω, respectively. Better performance was achieved with the Mn:ZnO electrode and M:FA electrolyte compatibility. Using metal salts has significant potential for PSC applications with its low resistance and high photocurrent charge carriers.

Cyclic stability, which is critical for supercapacitors, was assessed at a voltage of 1 V and a current density of 60 Ag^-^^1^ (Figure 9A). After 10,000 cycles, charge retention was found to be 67%, 117%, and 55% of the original capacitance for Cu:FA, Co:FA, and Fe:FA PSCs (Figure S16). The Co:FA PSC has seen a gradual increase, exceeding 100%. This is attributed to the full activation of the active materials and the increased penetration of the electrolyte into the electrode materials.^63^ Furthermore, after 10,000 cycles, Coulombic efficiency (CE %) values for all PSCs were found to be close to 100%. M:FA PSCs exhibited excellent 100% capacitance retention and Coulombic efficiency under light for 1000 cycles (Figure 9B). The capacitance retention response to light irradiation indicates that the electrode materials have high light sensitivity and good electrical conductivity.^64^ This stability in the presence of light is attributed to the production of photocharge carriers that enhance redox processes and increase charge transfer efficiency, thereby improving the charge storage process.^53,54^ Furthermore, CV measurements taken after 1000 cycles under light are shown in Figure S17. Reversibility and stability were observed in a window potential width of 0–1.1 V. It should be noted that M:FA PSCs exhibit good cycle performance and high stability both under light and in the absence of light. The cost-effectiveness, high stability, and durability of metal fatty acid salts under frequent charge–discharge cycles suggest that they may be suitable materials for high-performance energy storage PSC applications.

**Figure 9:**
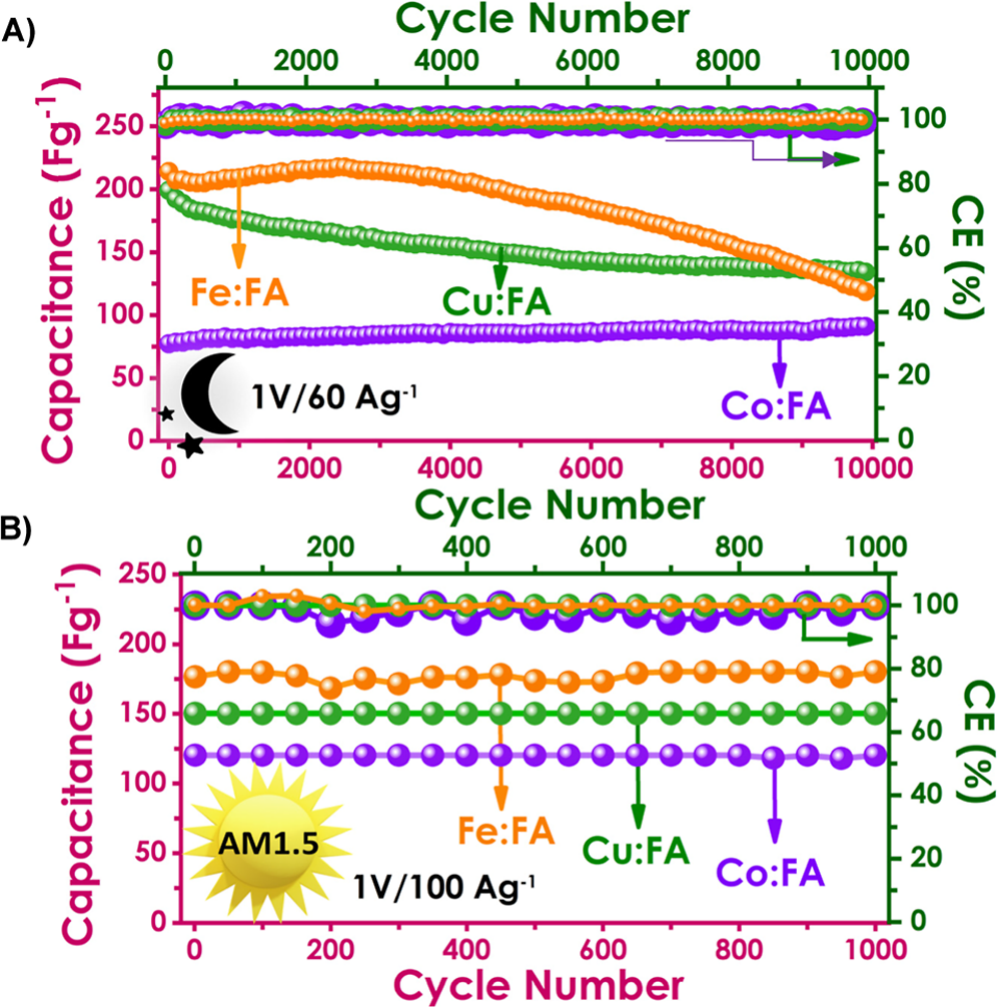
M:FA-based PSC devices: (A) cyclic stability (10,000) graphs in the dark and (B) cyclic stability (1000) graphs under AM1.5 light.

## 4 Conclusions

In this study, metal fatty acid-based gel electrolytes were developed for PSC studies. Three different metal-based (Co, Cu, and Fe) fatty acids were successfully incorporated in PVA for use as a gel electrolyte in a Mn:ZnONS photoactive electrode used in PSC devices. CV measurements indicated that the *C*_p_ of the Cu, Co, and Fe/FA-based PSC devices at 25 mVs^–1^ scan rate were enhanced by 17, 20, and 13% with AM1.5 illumination. The highest *C*_p_ of 381 Fg^-^^1^ has been calculated at a 25 mVs^–1^ scan rate for Cu:FA. As expected, a lower scan rate resulted in a higher *C*_p_. The highest *C*_p_, calculated using CV data taken at 1 mVs^–1^, was 644 Fg^-^^1^ for Fe:FA. However, considering all scanning rates, it was observed that the CV performances of PSC devices using gel electrolytes containing Cu:FA were higher than those containing Co and Fe. This result indicated that low-cost and Earth-abundant copper-based fatty acids can be integrated into PSC devices successfully. A similar result was obtained for GCD analysis. The energy and power densities of the Cu:FA-based PSC were superior to those of Co and Fe/FA-based devices. The highest energy and power densities of Cu/FA-based PSCs were 45 Whkg^–1^ and 30 kWkg^–1^, respectively. Although in terms of cyclic stability, Co/FA-based PSCs resulted in very high stability and 117% capacitance retention for 10,000 cycles, Cu/FA-based devices maintained 67% of their initial capacitance. At the same cycle number, Fe:FA showed relatively low cyclic stability, which is 55% compared to Co and Cu:FA. At the end, it is possible to conclude that M:FA-incorporated PVA gel electrolytes are very promising for lithium-free electrolytes, not only for photorechargeable supercapacitors but also for other electrochemical energy storage devices.

## Supplementary Material



## Data Availability

The data that support the findings of this study are available within the article and its Supporting Information. Additional raw data are part of an ongoing study and will be made available from the corresponding author upon reasonable request.

## Notes

SEM images and EDS spectra of Cu:FA, Co:FA, and Fe:FA powders (Figures S1–S3); BET analysis of Mn:ZnONS powder (Figure S4); SEM images and EDS of Mn:ZnONS powder and thin film (Figures S5 and S6); CV, *C*_p_, and GCD analysis of Cu:FA-, Co:FA-, and Fe/FA-based PSC devices (Figures S7–S9); CV comparison of M/FA-based PSC devices (Figure S10); GCD results for Cu:FA-, Co:FA-, and Fe/FA-based PSC devices (Tables S1–S3); Dunn’s method analysis of capacitive and diffusive contributions (Figures S11–S13); LSV graphs (Figure S14); EIS Nyquist plots and fitting results (Figure S15, Table S4); cyclic stability graphs in the dark and under AM1.5 (Figures S16 and S17); and literature comparison of ZnO-based PSCs and fatty acid SC performances (Table S5) (PDF)
