# Supplementary material for: Lithium-Free Metal Fatty Acid-Based Electrolytes for Quasi-Solid-State Photosupercapacitors
Source: ACS Omega. 2026 Apr 1;11(14):21770–80. doi: 10.1021/acsomega.5c10959 (PMC13084477; doi:10.1021/acsomega.5c10959)
Supplement: Supplementary file 1 [file ao5c10959_si_001.pdf]

Supporting Information

Lithium-Free Metal Fatty Acid-Based Electrolytes for Quasi-Solid State Photosupercapacitors

Emine Karagoz,[a] Cemre Oztop[b], Deren Aydin[b], Cigdem Tuc Altaf,[b,c] Nurdan Demirci Sankir [a, b] ‡, Mehmet Sankir [a, b] §

[a] Micro and Nanotechnology Graduate Program, TOBB University of Economics and Technology, Sogutozu Caddesi No 43 Sogutozu 06560 Ankara, Turkey

[b] Department of Materials Science and Nanotechnology Engineering, TOBB University of Economics and Technology, Sogutozu Caddesi No 43 Sogutozu 06560 Ankara, Turkey

[c] Fotontek Optical Communication Ltd. Sti., Aso Technopark Ahi Evran Osb, Erkunt Avenue No: 3 Office No: 114, 06935 Sincan/Ankara, Turkey

Corresponding authors: ‡ nsankir@etu.edu.tr. Phone: +90 312 2924331**.** Fax: +90 312 2924121. § msankir@etu.edu.tr. Phone: +90 312 2924332. Fax: +90 312 292 4121

Table of Contents

1. **Materials Characterizations………………………………………………………...4**

Figure S1. A-E) SEM images at different magnifications, F) Energy-dispersive X-ray spectroscopy (EDS) of Cu:FA, G) Photograph of Cu:FA powder………………………….5

Figure S2. A-E) SEM images at different magnifications, F) Energy-dispersive X-ray spectroscopy (EDS) of Co:FA, G) Photograph of Co:FA powder………………………….6

Figure S3. A-E) SEM images at different magnifications. F) Energy-dispersive X-ray spectroscopy (EDS) of FeFA, G) Photograph of Fe:FA powder……………………..….7

Figure S4. A), B) BET analysis results of Mn:ZnONS powder before 5 hours of sonication………………………………………………………..……………..8

Figure S5. A-E) SEM images of Mn:ZnONS powder at different magnifications. F) Energy-dispersive X-ray spectroscopy (EDS) of Mn:ZnONS powder, G) Photograph of Mn:ZnONS powder……………………………………………………………………………....9

Figure S6. A-F) SEM images of the Mn:ZnONS powder after sonication for 5 hours and the resulting thin film at different magnifications, G) Photograph of Mn:ZnO thin film on FTO substrate……………………………………………………………….……..10

2. **Electrochemical Measurements…………………………………………………11**

Figure S7. A) CV graph at different scanning rates and in dark; B) Without electrode (Mn:ZnONS) CV graph at different scanning rates and in dark; C) CV graph at different window potentials and in dark, D) CV graph at different window potentials and AM1.5; E) C_p graph at different scanning rates and in dark; F) GCD graph at different current densities and in the dark; G) GCD graph at different current densities and in the AM1.5 light of Cu:FA PSC…………………………………………………………………..………12

Figure S8. A) CV graph at different scanning rates and in dark; B) C_p graph at different scanning rates and in dark; C) CV graph at different window potentials and in dark, D) CV graph at different window potentials and AM1.5; E) GCD graph at different current densities and in the dark; F) GCD graph at different current densities and in the AM1.5 light of Co:FA PSC…………………………………………………………………………..…13

Figure S9. A) CV graph at different scanning rates and in dark; B) C_p graph at different scanning rates and in dark; C) CV graph at different window potentials and in dark, D) CV graph at different window potentials and AM1.5; E) GCD graph at different current densities and in the dark; F) GCD graph at different current densities and in the AM1.5 light of Fe:FA PSC……………………………………………………………………………..14

Figure S10. A) CV graph at 100 mVs^{-1} scanning rate and in dark; B) CV graph at 100 mVs^{-1} scanning rate and in AM1.5 light of M:FA-based PSC…………………….…15

Table S1. GCD analysis results for Cu:FA-based PSC device at dark and under illumination……………………………………………………………………..15

Table S2. GCD analysis results for Co:FA-based PSC device at dark and under illumination………………………………………………………………………..……16

Table S3. GCD analysis results for Fe:FA-based PSC device at dark and under illumination…………………………………………………………………………….…16

Figure S11. A), B) Variation of b-values as a function of potential in darkness and light, C), D) Power-law dependence of the peak current at scan rates from 1 to 100 mVs^{-1} in darkness and light; E), F) Deconvoluted percentage contribution of capacitive and diffusive current contribution at different scan rates of Cu:FA PSC………………………………………17

Figure S12. A), B) Variation of b-values as a function of potential in darkness and light, C), D) Power-law dependence of the peak current at scan rates from 1 to 100 mVs^{-1} in darkness and light; E), F) Deconvoluted percentage contribution of capacitive and diffusive current contribution at different scan rates of Co:FA PSC………………………………………18

Figure S13. A), B) Variation of b-values as a function of potential in darkness and light, C), D) Power-law dependence of the peak current at scan rates from 1 to 100 mVs^{-1} in darkness and light; E), F) Deconvoluted percentage contribution of capacitive and diffusive current contribution at different scan rates of Fe:FA PSC……………………………………….19
19

Figure S14. A) Cu:FA PSC linear sweep voltammetry (LSV) graph under dark and AM1.5 light, B) Co:FA PSC LSV graph under dark and AM1.5 light, C) Fe:FA PSC LSV graph under dark and AM1.5 light, D) Cu:FA, Co:FA and Fe:FA PSC LSV graphs under dark…20

Figure S15. A) EIS characteristics (Nyquist plots) of Cu:FA PSC with and without electrode, B) EIS characteristics (Nyquist plots) of Co:FA PSC with and without electrode, C) EIS characteristics (Nyquist plots) of Fe:FA PSC with and without electrode………..21

Table S4. EIS results for Cu:FA, Co:FA and Fe:FA-based PSC devices……………..…22

Figure S16. Capacitance retention graph for 10,000 measurement cycles for M:FA-based PSC devices in the dark…………………………………………………………..…..22

Figure S16. Capacitance retention graph for 10,000 measurement cycles for M:FA-based PSC devices in the dark………………………………………………………………22

Figure S17. A) Capacitance retention graph for 1000 measurement cycles for M:FA-based PSC devices in the AM1.5, B) CV graph of M:FA-based PSC devices after 1000 cycles under AM1.5 light at a scan rate of $25mVs^{-1}$…………………………………………...23

Table S5. Literature comparison of ZnO-based PSC device and fatty acid containing SC device performances……………………………………………………………………….24

3. References………………………………………………………………………..….24

1. Materials Characterizations

Scanning electron microscopy (SEM) and energy dispersive X-ray (EDS) results of Cu:FA, Co:FA, Fe:FA fatty acid powders and Mn:ZnONS thin film are presented below, along with the BET (Brunauer-Emmett-Teller) analysis result of the Mn:ZnONS thin film before sonication for 5 hours.

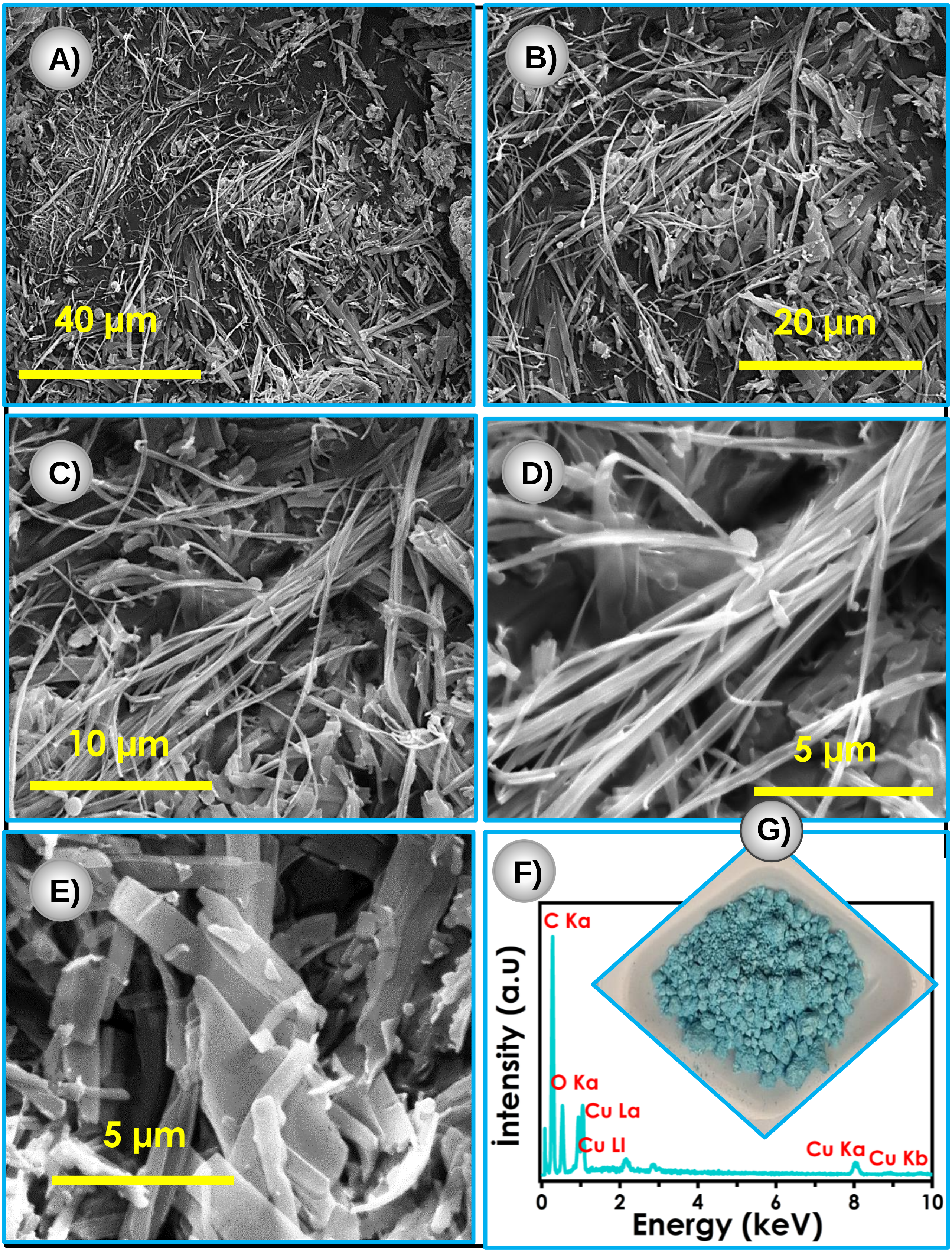

Figure S1. A-E) SEM images at different magnifications, **F)** Energy-dispersive X-ray spectroscopy (EDS) of Cu:FA, **G)** Photograph of Cu:FA powder.

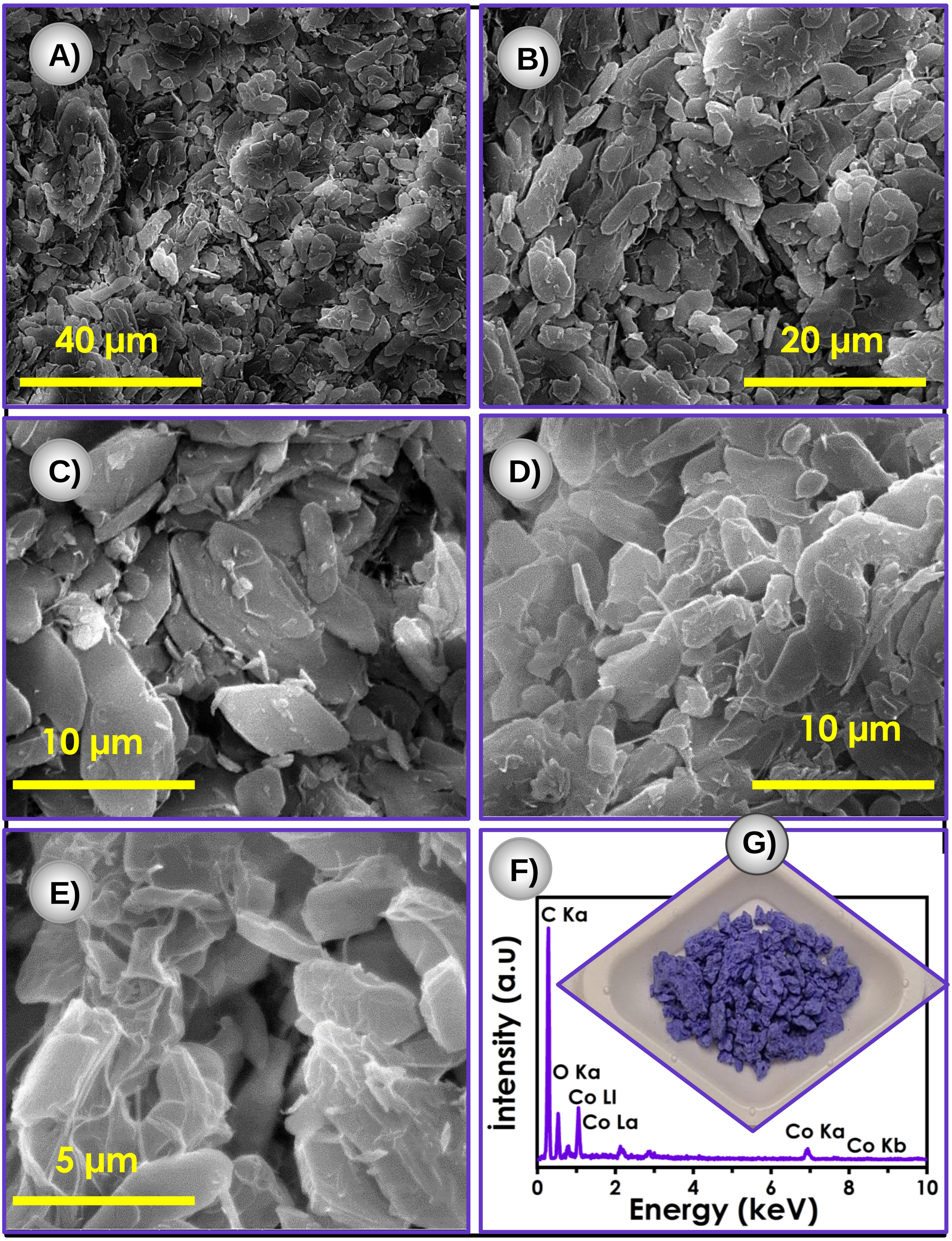

Figure S2. A-E) SEM images at different magnifications, **F)** Energy-dispersive X-ray spectroscopy (EDS) of Co:FA, **G)** Photograph of Co:FA powder.

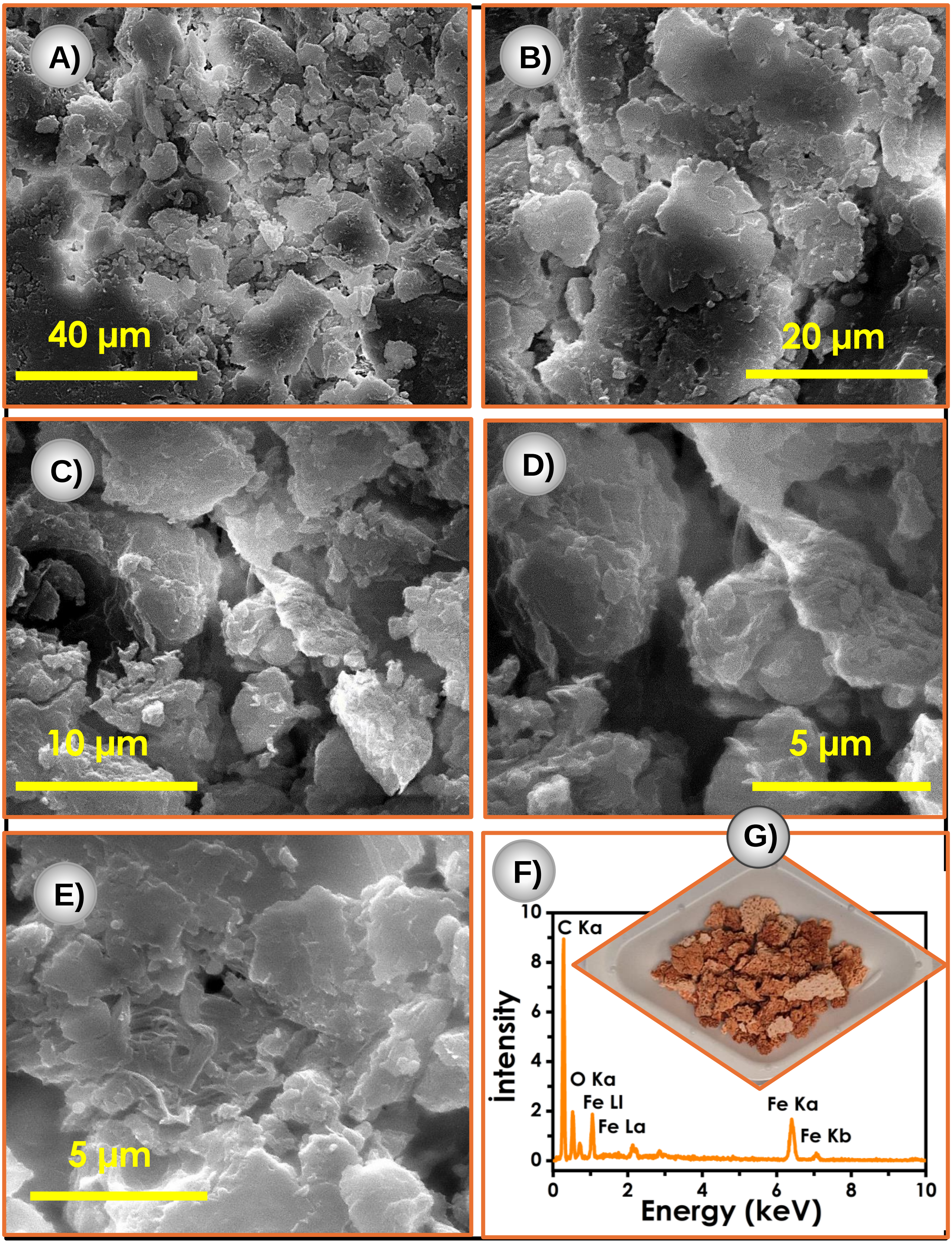

Figure S3. A-E) SEM images at different magnifications. **F)** Energy-dispersive X-ray spectroscopy (EDS) of FeFA, **G)** Photograph of Fe:FA powder.

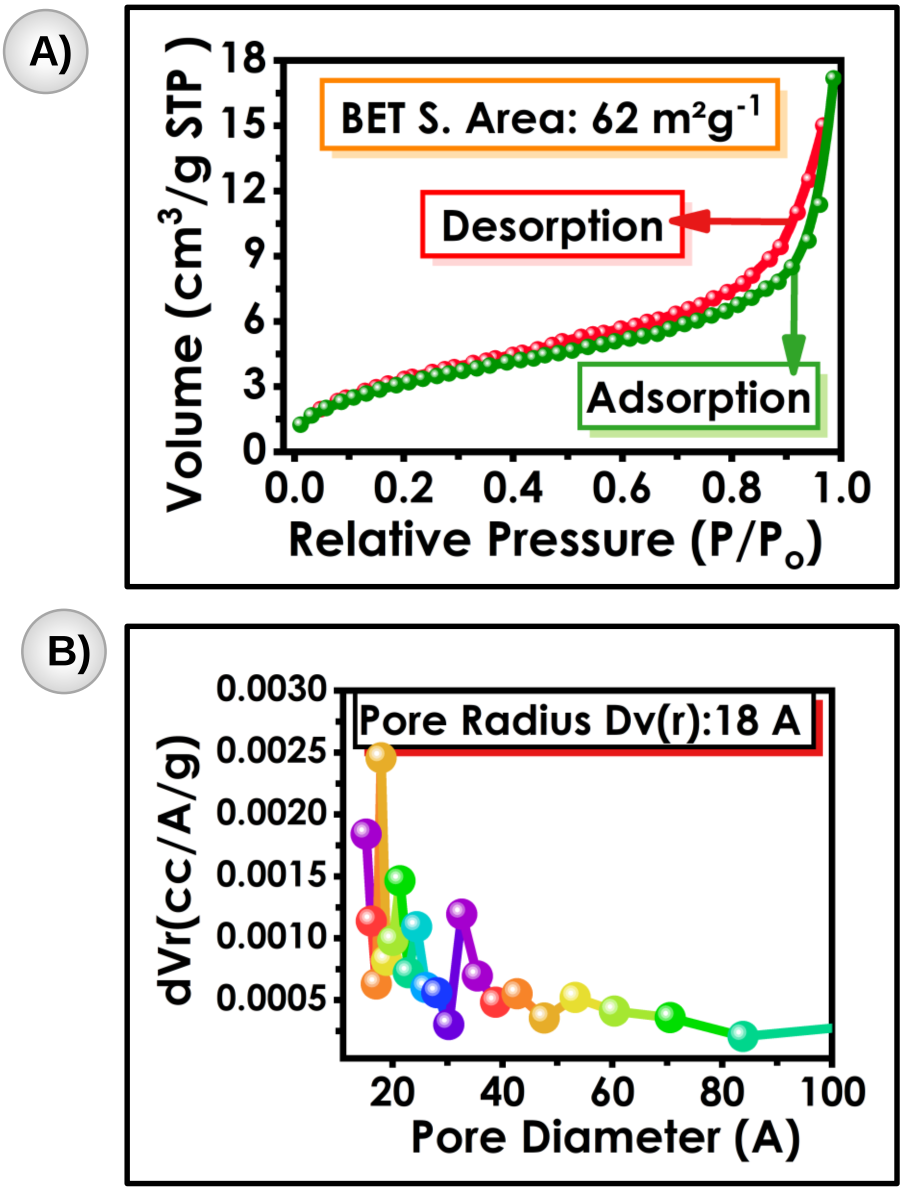

Figure S4. A), B) BET analysis results of Mn:ZnONS powder before 5 hours of sonication.

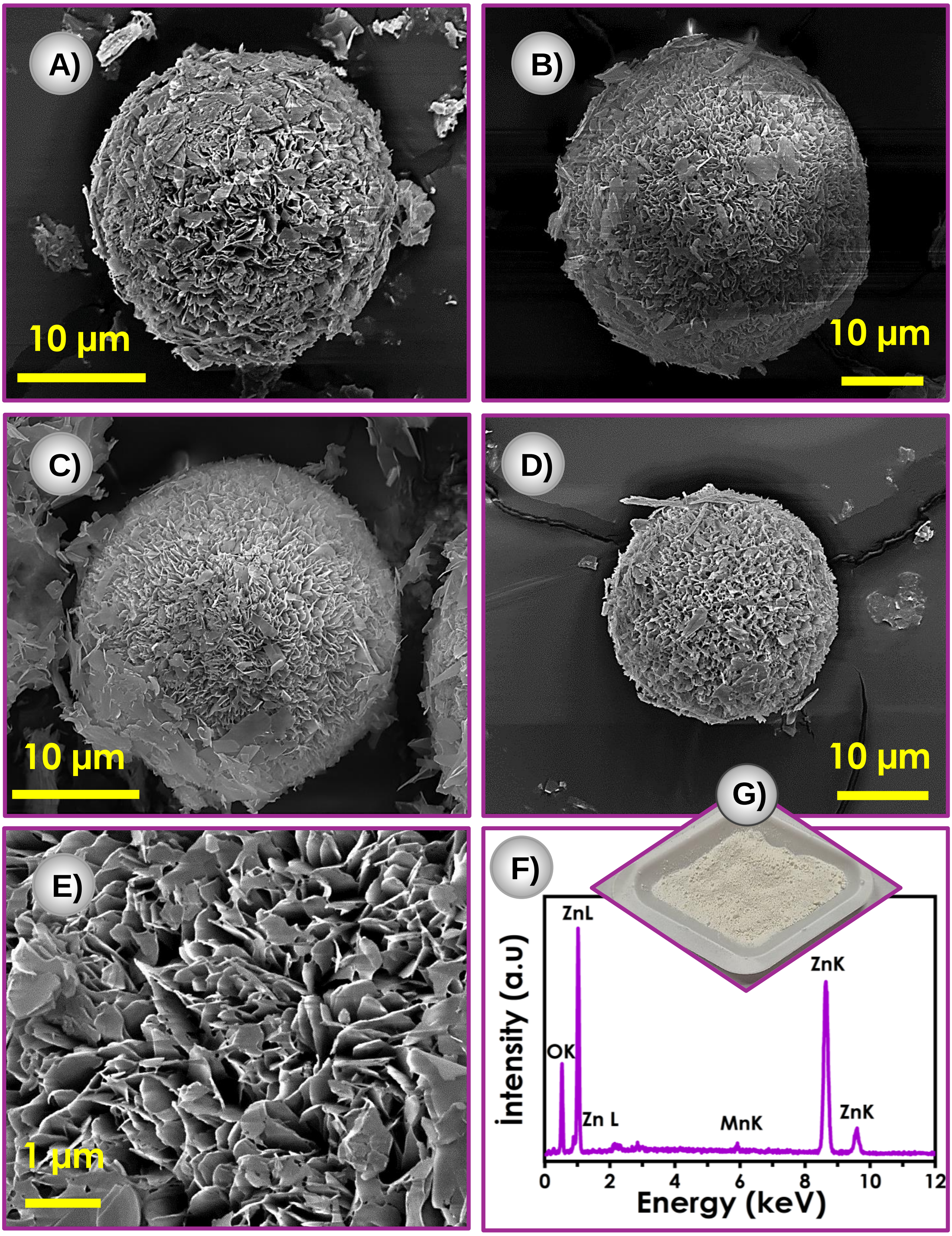

Figure S5. A-E) SEM images of Mn:ZnONS powder at different magnifications. **F)** Energy-dispersive X-ray spectroscopy (EDS) of Mn:ZnONS powder, **G)** Photograph of Mn:ZnONS powder.

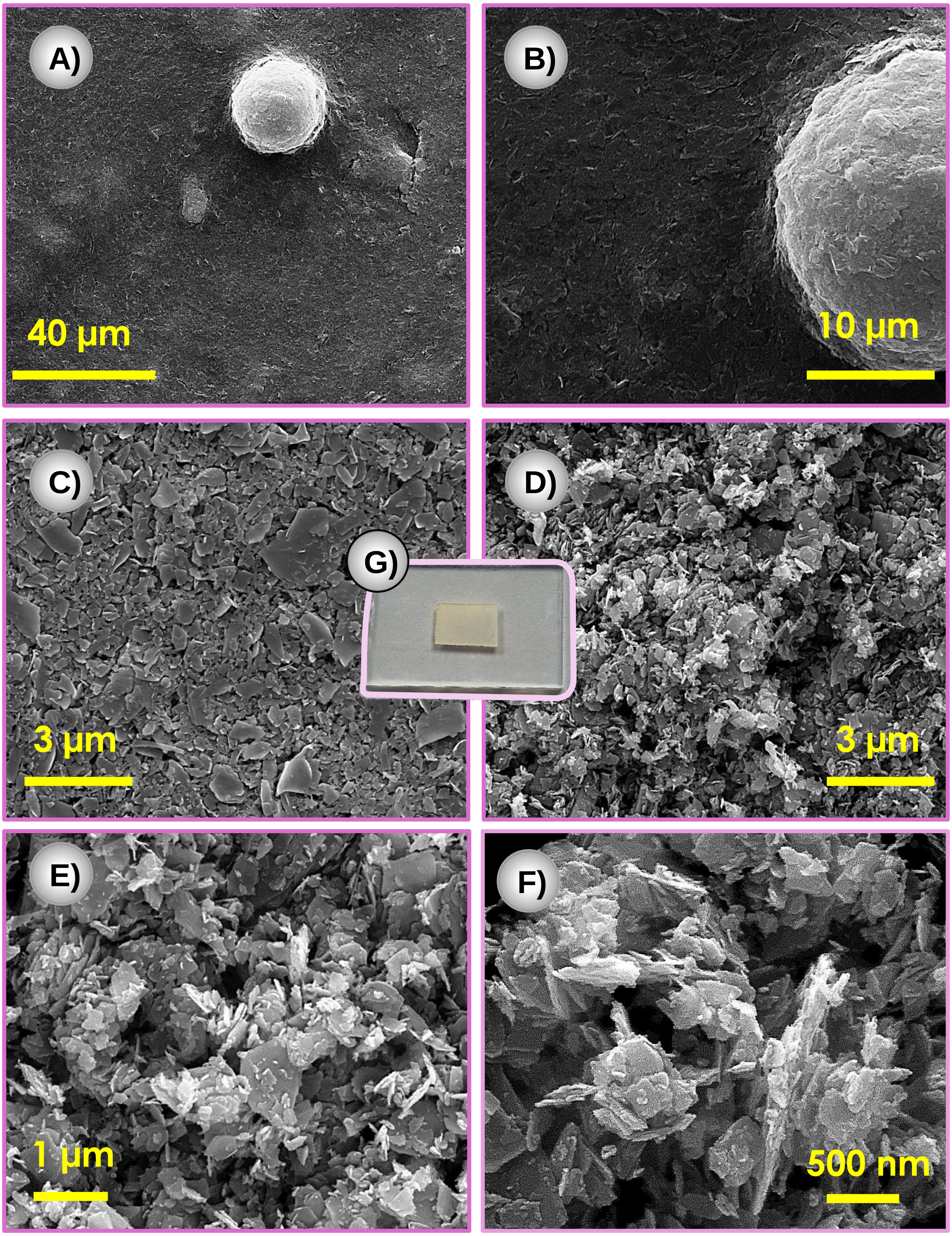

Figure S6. A-F) SEM images of the Mn:ZnONS powder after sonication for 5 hours and the resulting thin film at different magnifications, **G)** Photograph of Mn:ZnO thin film on FTO substrate.

2. Electrochemical Measurements

Detailed cyclic voltammetry (CV), specific capacitance (C_p), galvanostatic charge-discharge (GCD), Dunn's test (Capacitive and diffusive contributions), linear sweep voltammetry (LSV), electrochemical impedance spectroscopy (EIS), and cyclic stability (capacitance retention) results, obtained from dark and light measurements of Cu:FA, Co:FA, and Fe:FA PSCs produced using different fatty acids, are presented below.

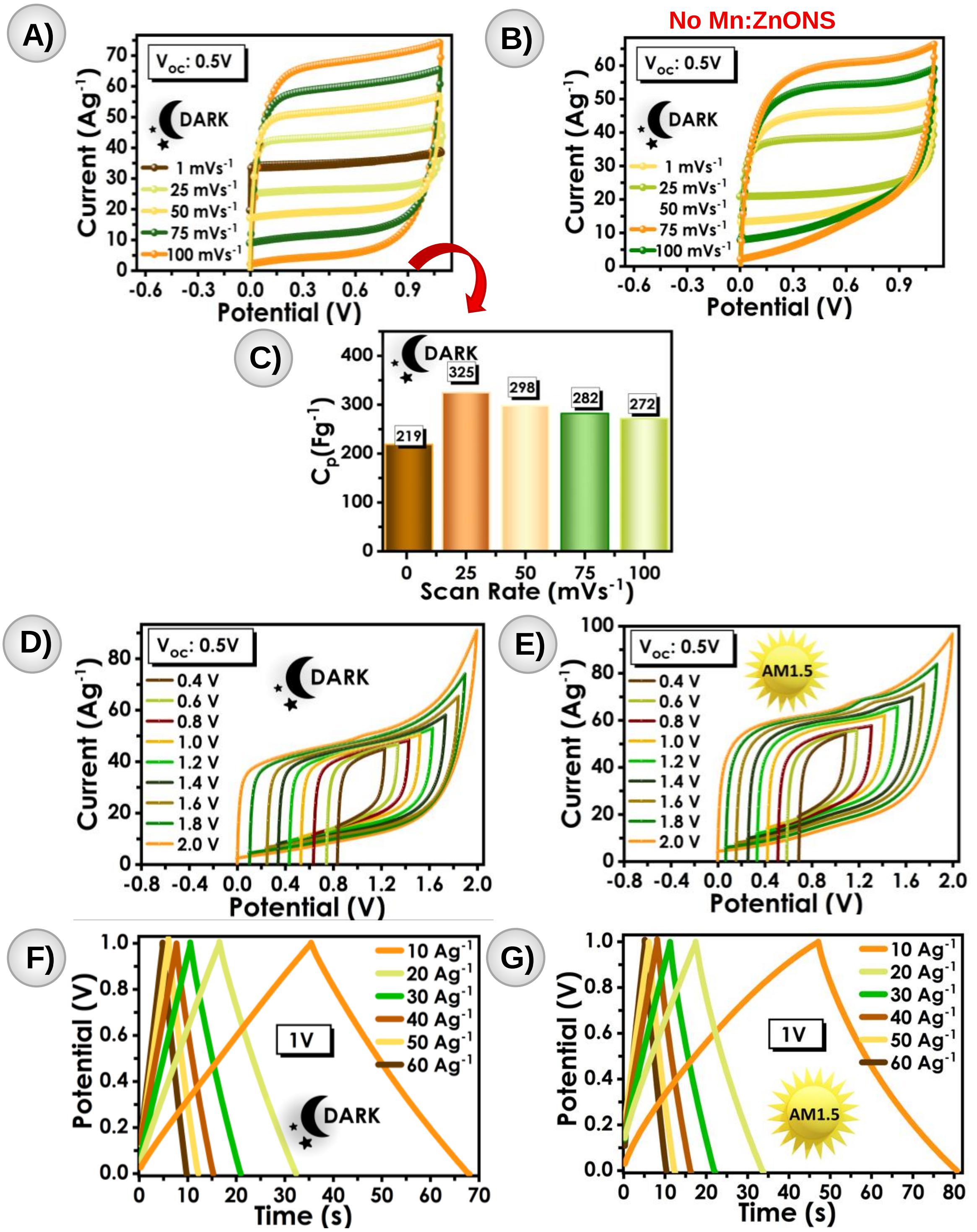

Figure S7. A) CV graph at different scanning rates and in dark; **B)** Without electrode (Mn:ZnONS) CV graph at different scanning rates and in dark; **C)** CV graph at different window potentials and in dark, **D)** CV graph at different window potentials and AM1.5; **E)** C_p graph at different scanning rates and in dark; **F)** GCD graph at different current densities and in the dark; **G)** GCD graph at different current densities and in the AM1.5 light of Cu:FA PSC.

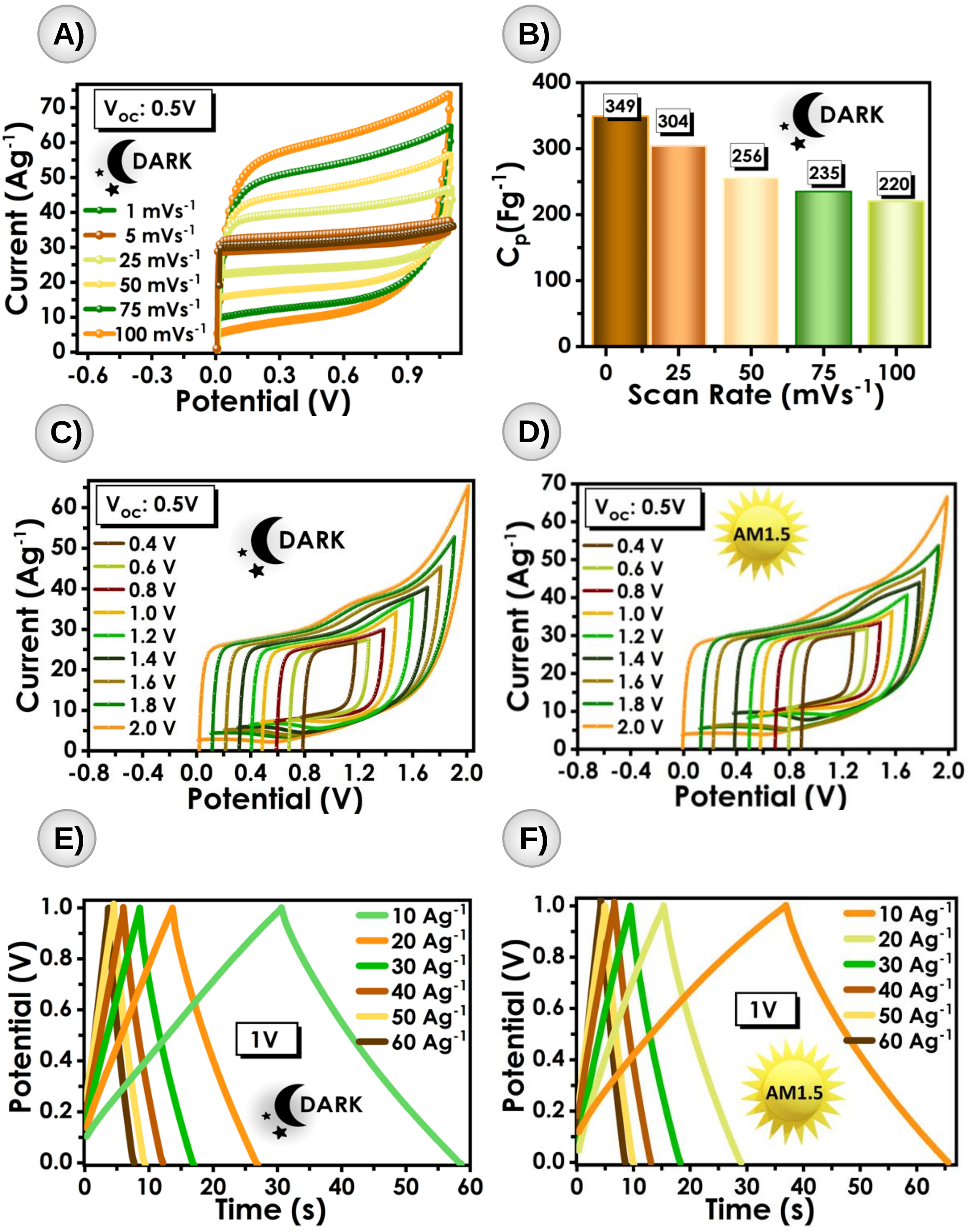

Figure S8. A) CV graph at different scanning rates and in dark; **B)** C_p graph at different scanning rates and in dark; **C)** CV graph at different window potentials and in dark, **D)** CV graph at different window potentials and AM1.5; **E)** GCD graph at different current densities and in the dark; **F)** GCD graph at different current densities and in the AM1.5 light of Co:FA PSC.

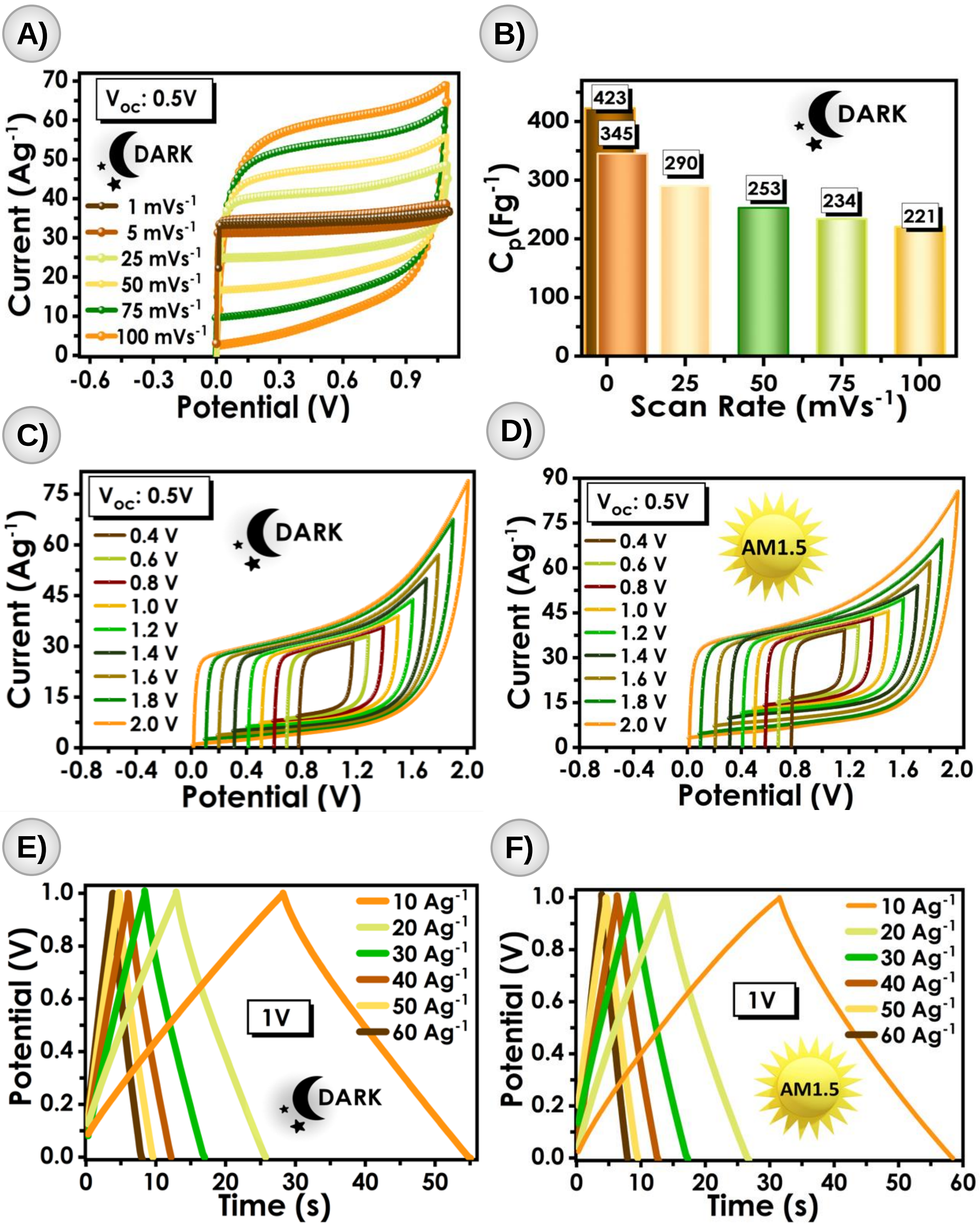

Figure S9. A) CV graph at different scanning rates and in dark; **B)** C_p graph at different scanning rates and in dark; **C)** CV graph at different window potentials and in dark, **D)** CV graph at different window potentials and AM1.5; **E)** GCD graph at different current densities and in the dark; **F)** GCD graph at different current densities and in the AM1.5 light of Fe:FA PSC.

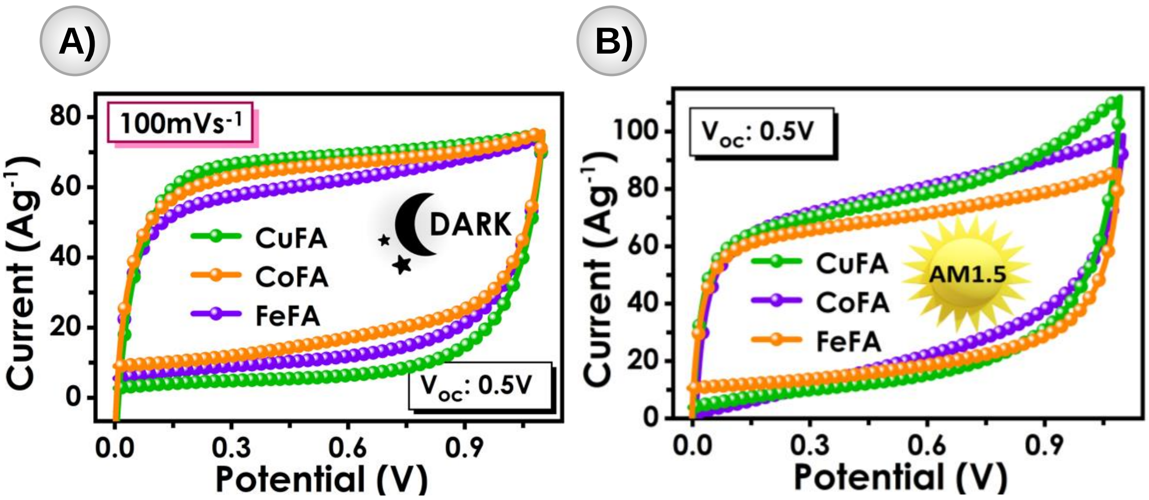

Figure S10. A) CV graph at 100 mVs^{-1} scanning rate and in dark; **B)** CV graph at 100 mVs^{-1} scanning rate and in AM1.5 light of M:FA-based PSC.

Table S1. GCD analysis results for Cu:FA-based PSC device at dark and under illumination

**Cu:FA-DARK**							
**t_c (s)**	**t_d (s)**	**V(V)**	**I (Ag^{-1})**	**CE%**	**C_p (F/g)**	**E_d (Wh/kg)**	**P(W/kg)**
4.8	4.8	1	60	100	288	40	30000
6.0	5.9	1	50	97	293	41	25000
7.7	7.4	1	40	96	295	41	20000
10.5	10.1	1	30	96	302	42	15000
16.5	15.6	1	20	94	311	43	10000
**35.4**	**32.1**	**1**	**10**	**91**	**321**	**45**	**5000**
**Cu:FA-AM1.5**							
**t_c (s)**	**t_d (s)**	**V(V)**	**I (Ag^{-1})**	**CE%**	**C_p (F/g)**	**E_d (Wh/kg)**	**P(W/kg)**
5.1	5.0	1	60	98	301	42	30000
6.1	6.0	1	50	99	300	42	25000
8.1	7.8	1	40	96	312	43	20000
11.2	10.5	1	30	94	315	44	15000
17.4	15.9	1	20	91	318	44	10000
**47.1**	**33.6**	**1**	**10**	**71**	**336**	**47**	**5000**

Table S2. GCD analysis results for Co:FA-based PSC device at dark and under illumination.

**CoFA-DARK**							
**t_c (s)**	**t_d (s)**	**V(V)**	**I (Ag^{-1})**	**CE%**	**C_p (F/g)**	**E_d (Wh/kg)**	**P(W/kg)**
3.7	3.6	1	60	97	218	30	30000
4.5	4.5	1	50	100	225	31	25000
6.0	5.7	1	40	96	230	32	20000
8.6	8.1	1	30	94	243	34	15000
13.7	12.9	1	20	95	258	36	10000
**30.6**	**27.6**	**1**	10	**90**	**276**	**38**	**5000**
**CoFA-AM1.5**							
**t_c (s)**	**t_d (s)**	**V(V)**	**I (Ag^{-1})**	**CE%**	**C_p (F/g)**	**E_d (Wh/kg)**	**P(W/kg)**
4.2	4.1	1	60	97	245	34	30000
5.0	4.8	1	50	97	240	33	25000
6.6	6.2	1	40	94	247	34	20000
9.4	8.5	1	30	90	256	36	15000
15.3	13.2	1	20	87	265	37	10000
**36.9**	**28.5**	**1**	10	**77**	**285**	**40**	**5000**

Table S3. GCD analysis results for Fe:FA-based PSC device at dark and under illumination.

**Fe:FA-DARK**							
**t_c (s)**	**t_d (s)**	**V(V)**	**I (Ag^{-1})**	**CE%**	**C_p (F/g)**	**E_d (Wh/kg)**	**P(W/kg)**
3.9	3.9	1	60	100	234	33	30000
4.8	4.7	1	50	99	236	33	25000
6.0	5.9	1	40	98	237	33	20000
8.4	8.1	1	30	96	243	34	15000
12.9	12.4	1	20	96	249	35	10000
28.2	26.4	1	10	94	264	37	5000
**Fe:FA-AM1.5**							
**t_c (s)**	**t_d (s)**	**V(V)**	**I (Ag^{-1})**	**CE%**	**C_p (F/g)**	**E_d (Wh/kg)**	**P(W/kg)**
3.9	3.9	1	60	100	234	32	30000
4.6	4.6	1	50	100	232	32	25000
6.3	6.0	1	40	95	240	33	20000
8.7	8.1	1	30	93	243	34	15000
13.8	12.6	1	20	91	252	35	10000
31.5	26.6	1	10	84	266	37	5000

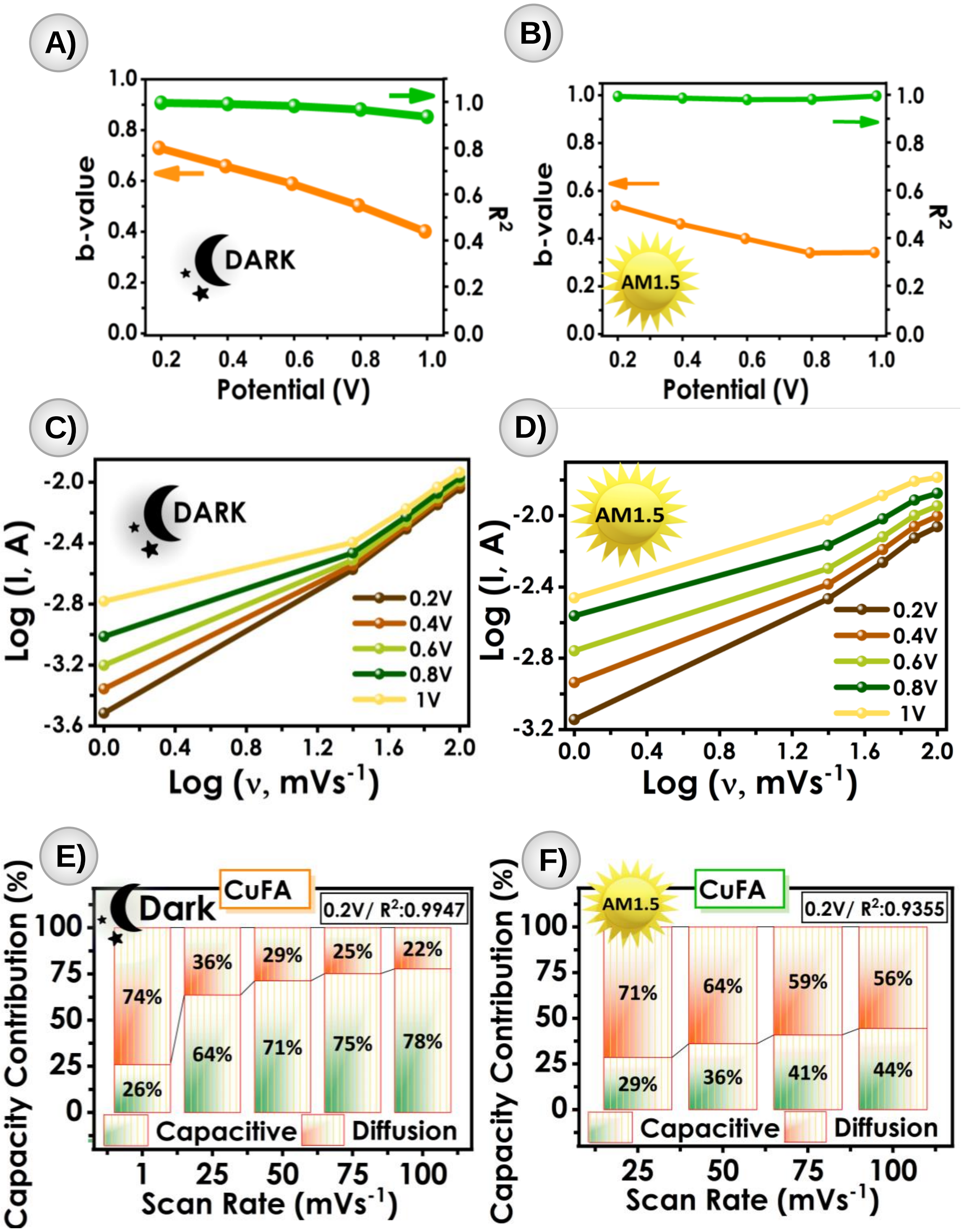

Figure S11. A), B) Variation of b-values as a function of potential in darkness and light, **C), D)** Power-law dependence of the peak current at scan rates from 1 to 100 mVs^{-1} in darkness and light; **E), F)** Deconvoluted percentage contribution of capacitive and diffusive current contribution at different scan rates of Cu:FA PSC.

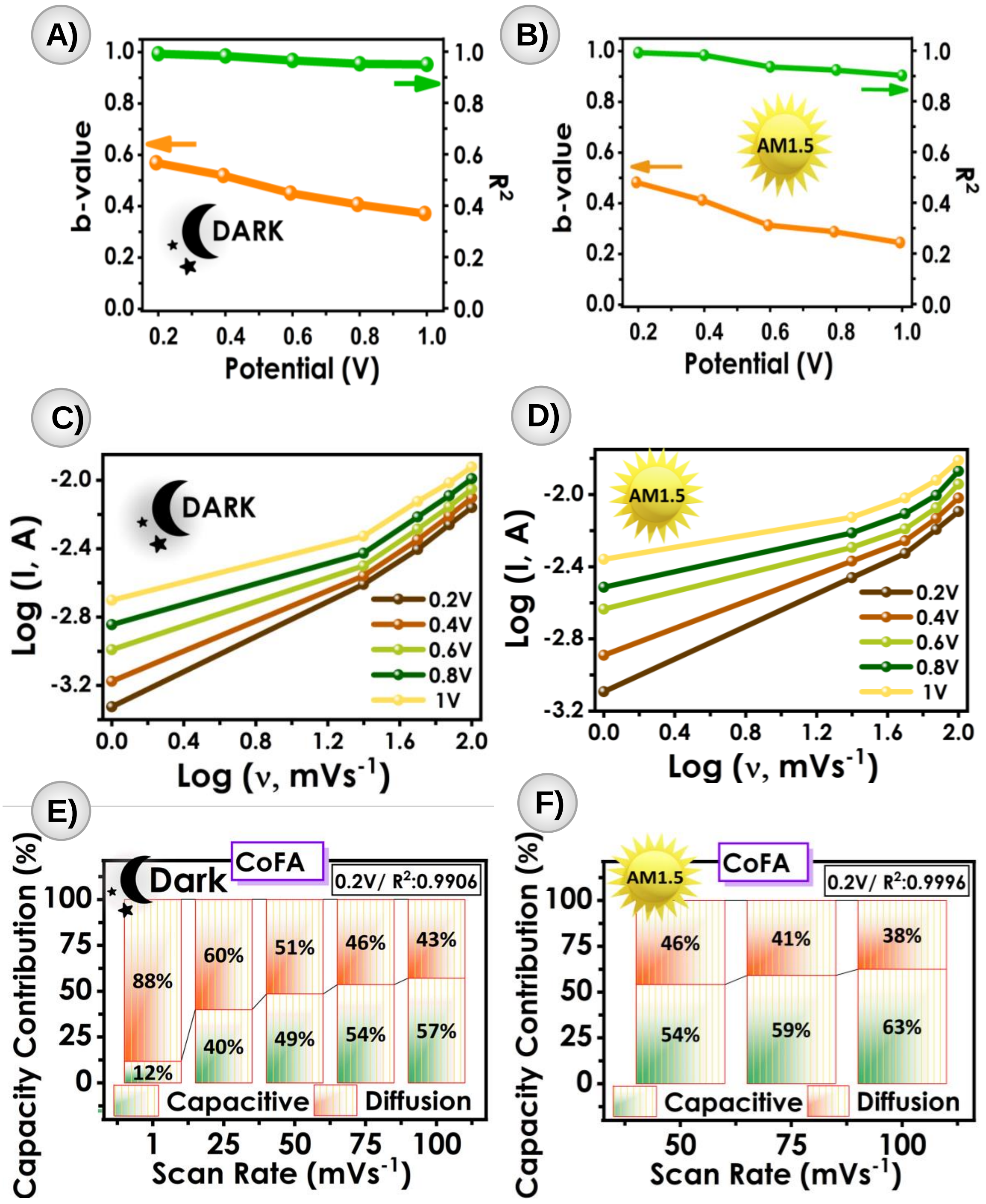

Figure S12. A), B) Variation of b-values as a function of potential in darkness and light, **C), D)** Power-law dependence of the peak current at scan rates from 1 to 100 mVs^{-1} in darkness and light; **E), F)** Deconvoluted percentage contribution of capacitive and diffusive current contribution at different scan rates of Co:FA PSC.

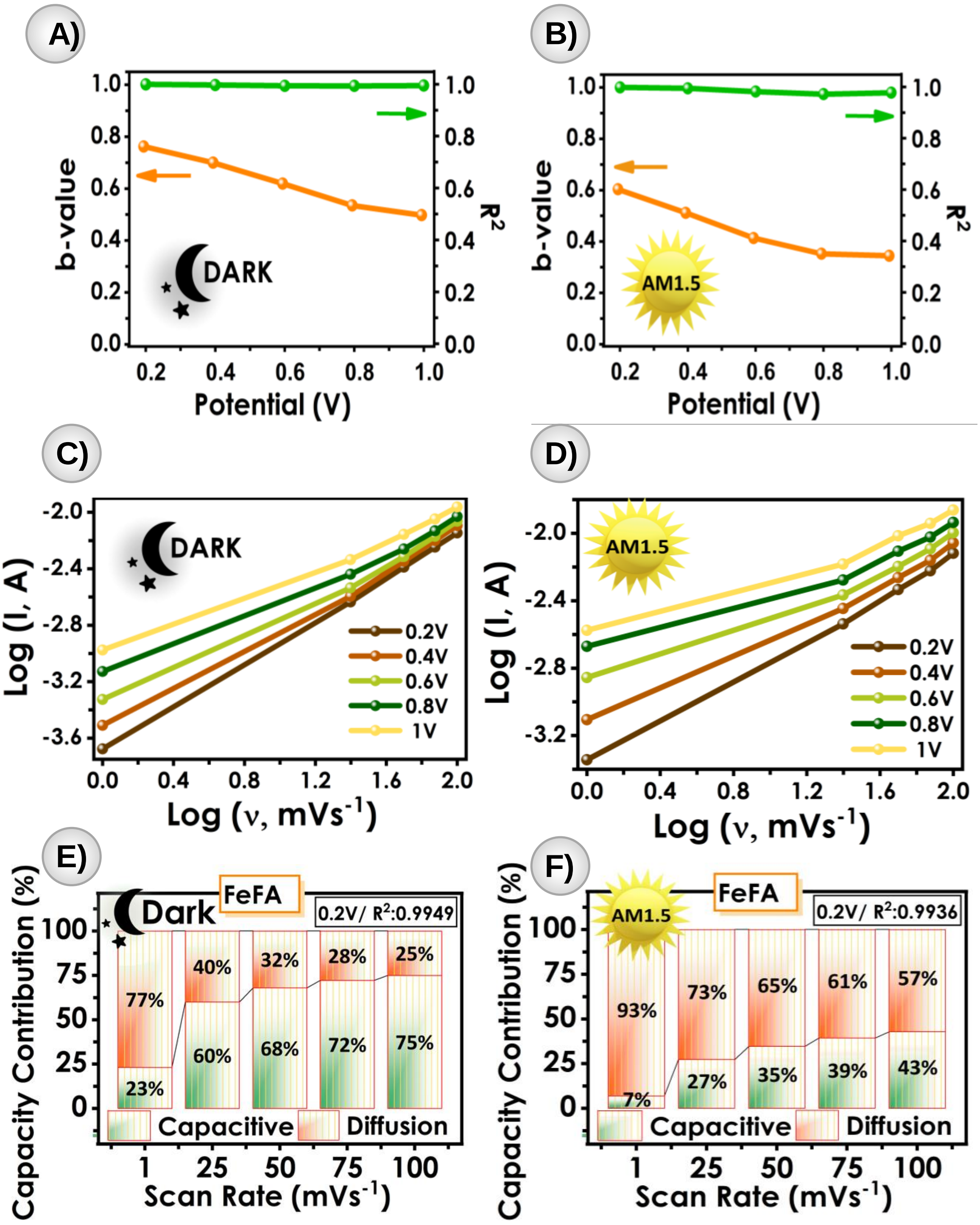

Figure S13.A), B) Variation of b-values as a function of potential in darkness and light, **C), D)** Power-law dependence of the peak current at scan rates from 1 to 100 mVs^{-1} in darkness and light; **E), F)** Deconvoluted percentage contribution of capacitive and diffusive current contribution at different scan rates of Fe:FA PSC.

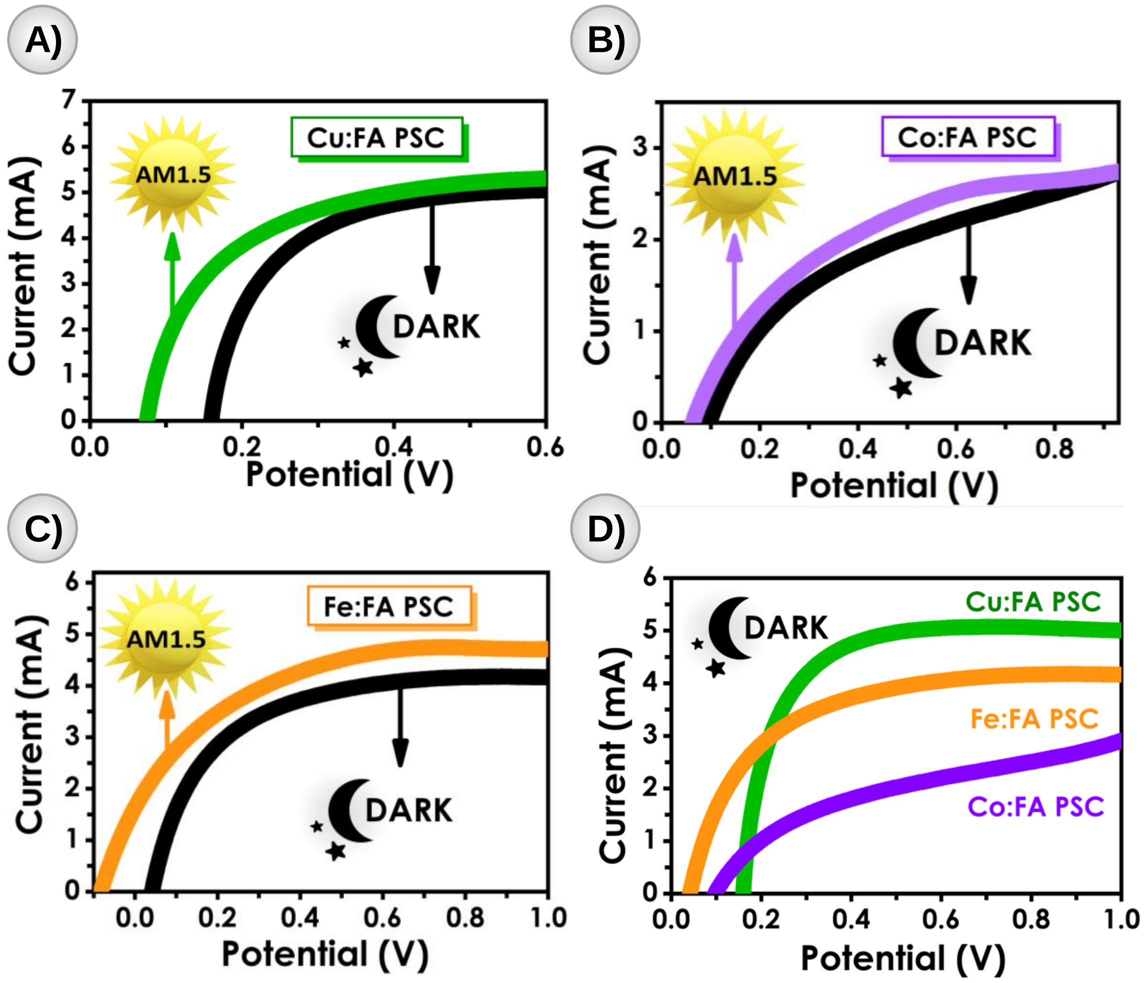

Figure S14 A) Cu:FA PSC linear sweep voltammetry (LSV) graph under dark and AM1.5 light, **B)** Co:FA PSC LSV graph under dark and AM1.5 light, **C)** Fe:FA PSC LSV graph under dark and AM1.5 light, **D)** Cu:FA, Co:FA and Fe:FA PSC LSV graphs under dark.

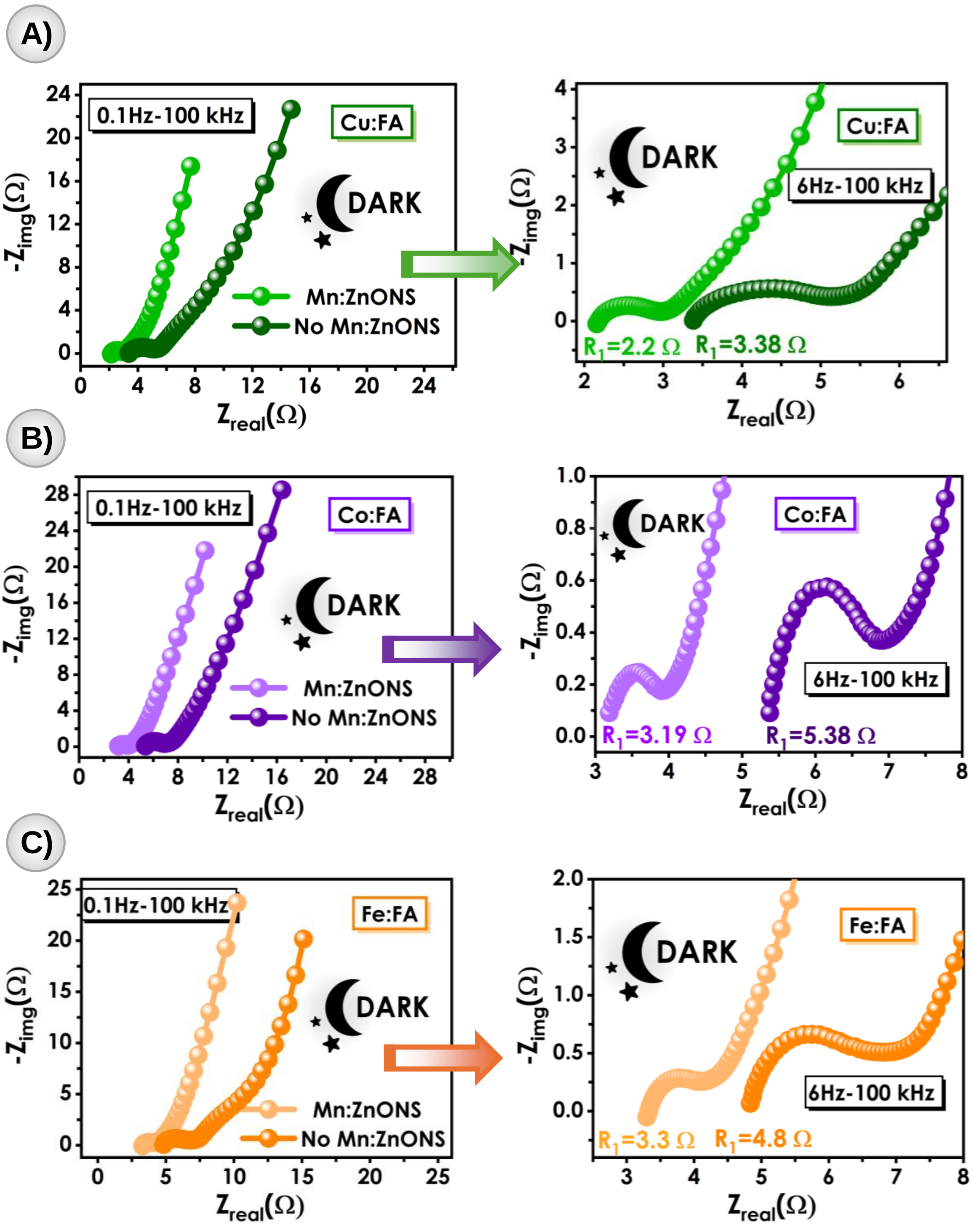

Figure S15. A) EIS characteristics (Nyquist plots) of Cu:FA PSC with and without electrode, **B)** EIS characteristics (Nyquist plots) of Co:FA PSC with and without electrode, **C)** EIS characteristics (Nyquist plots) of Fe:FA PSC with and without electrode.

Table S4. EIS results for Cu:FA, Co:FA and Fe:FA-based PSC devices.

PSC Device	R1 (Ω)	C1 (µF)	R2 (Ω)	C1 (F)	R2 (Ω)	s($\Omega s^{-1/2}$)
Cu:FA&Dark	2.18	30	0.75	0.125	1600	3.75
Cu:FA&AM1.5	**2**	**30**	**0.74**	**0.122**	**900**	**3.75**
Co:FA&Dark	3.2	30	0.7	0.096	300	4.3
Co:FA&AM1.5	**2.6**	**30**	**0.55**	**0.096**	**250**	**3.75**
Fe:FA&Dark	3.3	30	0.8	0.09	600	4.4
Fe:FA&AM1.5	**2.7**	**30**	**0.8**	**0.095**	**400**	**3.4**

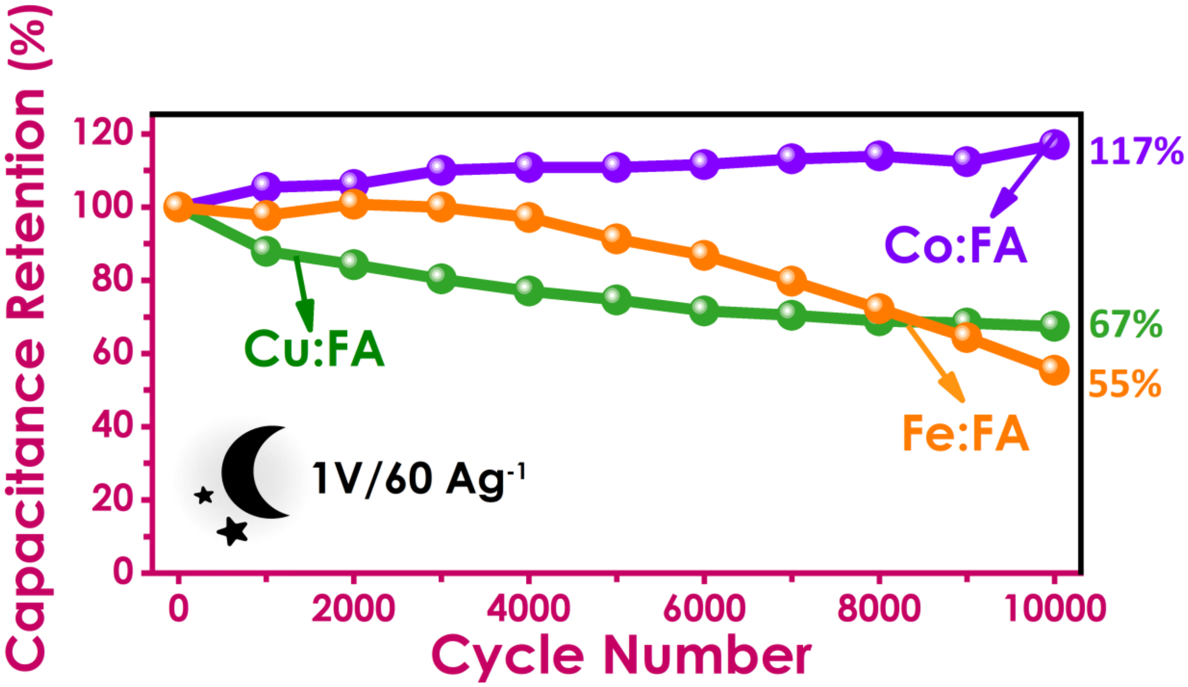

Figure S16. Capacitance retention graph for 10,000 measurement cycles for M:FA-based PSC devices in the dark.

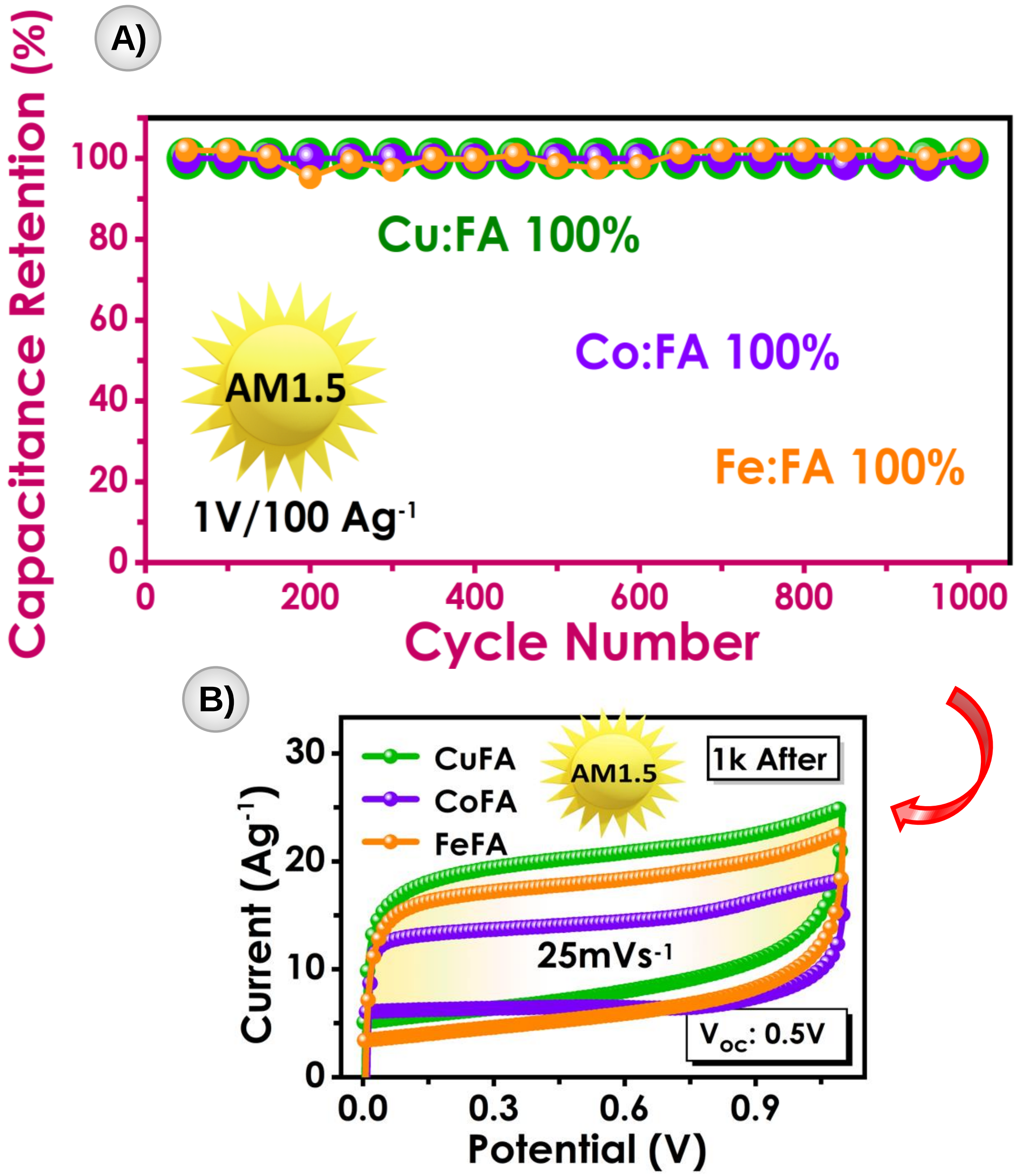

Figure S17. A) Capacitance retention graph for 1000 measurement cycles for M:FA-based PSC devices in the AM1.5, **B)** CV graph of M:FA-based PSC devices after 1000 cycles under AM1.5 light at a scan rate of $25mVs^{-1}$.

Table S5. Literature comparison of ZnO-based PSC device and fatty acid containing SC device performances.

**Cathode/ Electrode /Electrolyte/Anode**	**Potential**	**Specific Capacitance**	**Energy Density**	**Power Density**	**Cycles**	**Ref.**
Mn:ZnONS// PVA-Cu:FA// Activated Carbon	0-1.1 V	219 Fg^{-1}	45 Wh/kg	5 (kW/kg)	10,000& 67%	This Work &Dark
		559 Fg^{-1}	47 Wh/kg	5 (kW/kg)	1000& 100%	This Work &AM1.5
ZnONF// PVA/LiCl// Activated Carbon	1.99 V	1.98 mF g^{-1}	3.4mWh/kg	-	3000& 100%	[1]&Dark
		4.15 mF g^{-1}	8.3mWh/kg	-		[1]&UV
ZnO//CdS// $BMIMBF_4$// FTO	0-1 V	88,8 µF cm^{-2}	-	-	5000& 94.7%	[2]&Dark
		6,5 µF cm^{-2}	1.08nWhcm^{-2}	1.08µWcm^{-2}	-	[2]&UV
Activated carbon electrodes//Na-based eutectic systems as electrolyte	0-1 V	71.6 Fg^{-1}	-	-	5000&92 %	Only SC device no light effect [3]

3. References

[1] Altaf CT, Coskun O, Kumtepe A, et al. Photo-supercapacitors based on nanoscaled ZnO. *Sci Rep*. 2022,12(1):1-15.

[2] Mahapatra A Das, Kumar S, Sutradhar A, Sahoo S, Misra A. ZnO/CdS based high performance broadband photo-chargeable flexible supercapacitor. *Electrochim Acta*. 2024,474,143507.

[3] Silva IG, Anouti M, Montemor M F, Marrucho I M, Natural Eutectic Mixtures of Sodium Salt and Fatty Acids as Electrolytes for Supercapacitors, ACS Sustainable Chem. Eng. 2024, 12, 1365−1377
